# Effect of Alkyl Chain Length on Physicochemical and Pharmacokinetic Performance of Aripiprazole Fatty Acid Prodrugs for Long-Acting Injectable Suspensions

**DOI:** 10.3390/pharmaceutics18080911

**Published:** 2026-07-24

**Authors:** An Chen, Cong Lai, Hao Zhai, Shiyang Zhang, Yijing Zhang, Ting Cai

**Affiliations:** 1 State Key Laboratory of Natural Medicines, Department of Pharmaceutical Engineering, School of Engineering, China Pharmaceutical University, Nanjing 211198, China; 2 Department of Pharmaceutics, School of Pharmacy, China Pharmaceutical University, Nanjing 211198, China

**Keywords:** prodrug, long-acting injectable aqueous suspensions, aripiprazole, fatty acid, chain length

## Abstract

**Background/Objectives**: Long-acting injectable aqueous suspensions based on fatty acid prodrugs offer a compelling strategy for chronic disease management. However, the selection of optimal alkyl chain length remains largely empirical, as its influence on prodrug performance is highly system-dependent and lacks predictive guidelines. **Methods**: Three aripiprazole prodrugs with different alkyl chain lengths were synthesized and characterized using ^1^H-NMR spectroscopy and single-crystal X-ray diffraction. A series of physicochemical assessments was performed, including melting point, solubility, lipophilicity, solid-state stability, and plasma stability. The prodrugs were subsequently formulated as aqueous suspensions, which were evaluated for particle size, morphology, release behavior, cytotoxicity, and cellular uptake. Finally, intramuscular administration in rats was carried out to investigate pharmacokinetic profiles and local tolerability. **Results**: Physicochemical characterization revealed that chain elongation progressively reduced the melting point, solubility, wettability, and solid-state stability of these prodrugs, whereas their flexibility, lipophilicity, and plasma stability correspondingly increased. After wet milling and intramuscular administration in rats, all suspensions sustained drug release for up to one month with good tolerability. Notably, the aripiprazole lauroxil formulation exhibited superior bioavailability and a shortened subtherapeutic interval, enabling the rapid attainment of therapeutic concentrations while effectively mitigating a prolonged pharmacokinetic tail. **Conclusions**: These findings reinforce the favorable profile of aripiprazole lauroxil as a lead candidate and demonstrate that isostructural packing facilitates the reliable prediction of chain length–property correlations across diverse fatty acid-prodrug systems, thus providing a valuable reference for rational alkyl chain selection in the design of fatty acid-based LAI suspensions.

## 1. Introduction

Poor medication adherence remains a major barrier in the long-term management of chronic diseases such as HIV/AIDS, schizophrenia, contraception, and diabetes [1]. Long-acting injectables (LAIs) represent a promising drug delivery platform to address this challenge [2]. They enable sustained drug release over weeks to months from a single injection, reducing dosing frequency, plasma drug fluctuations, and the incidence of adverse events while significantly enhancing patient adherence and reducing relapse risk [3]. Several LAI formulation platforms are now clinically established and commercially available, including microspheres, aqueous suspensions, in situ-forming gels, oil-based solutions, and implants [4,5]. Among these, long-acting injectable aqueous suspensions have garnered considerable attention from the pharmaceutical industry owing to their relatively high drug loading capacity and simple manufacturing process [2,6,7].

Fatty-acid prodrugs, by covalently attaching a fatty acid moiety to the parent drug, significantly reduce aqueous solubility and thus enable sustained release, rendering them a prevalent strategy for the development of long-acting injectable aqueous suspensions [8,9,10,11]. However, the choice of alkyl chain length for fatty-acid prodrugs in these formulations involves various factors that must be taken into account [12]. For instance, aripiprazole lauroxil was reported to have a high melting point and was selected for clinical studies, whereas paliperidone palmitate was identified based on its optimal pharmacokinetics and tolerability [12,13]. Meanwhile, among pipeline prodrug candidates, medium-chain brexpiprazole laurate and rilpivirine myristate have been advanced based on their favorable physicochemical properties and preliminary biopharmaceutical performance, respectively [14,15]. These examples highlight that optimal chain length selection remains system-dependent, a phenomenon attributed to the distinct crystal packing arrangements observed across alkyl chain homologues [12]. Therefore, systematic solid-state investigations of prodrugs across a range of chain lengths are essential for establishing the structure–property relationships [16].

**Figure 1:**
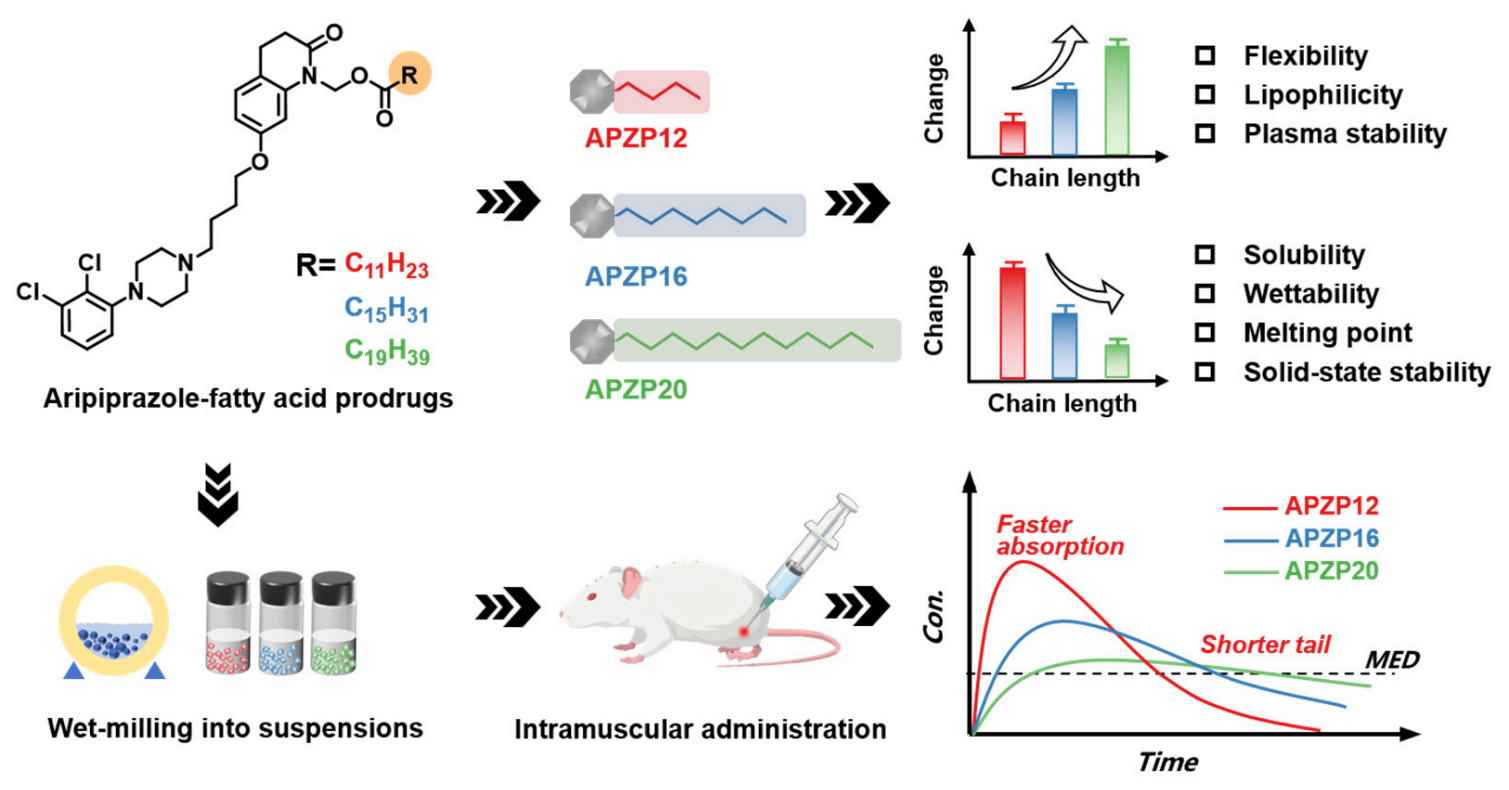
Chain length-dependent physicochemical and pharmacokinetic properties of aripiprazole-fatty acid prodrugs for long-acting injectable aqueous suspensions. Several elements in the image were obtained from BioGDP.com [21].

Aripiprazole lauroxil (APZP12) is an *N*-acyloxymethyl prodrug of aripiprazole with demonstrated efficacy and tolerability in treating schizophrenia [17,18]. Following administration, it undergoes a two-step bioconversion into active aripiprazole, which enters the systemic circulation to act as partial agonist activity at dopamine D2 and serotonin 5-HT1A receptors, and as an antagonist at serotonin 5-HT2A receptors [19,20]. Currently, there are two long-acting injectable aqueous suspensions of APZP12 approved by the U.S. FDA, developed and commercialized by Alkermes Pharmaceuticals as Aristada^®^ and Aristada Initio^®^, administered at monthly or six-week intervals. Despite the commercial preference for lauric acid in prodrug design, systematic comparisons with other homologous lipid chains remain limited, with the existing literature largely emphasizing its high melting point without addressing other potential advantages [12]. To probe the rationale beyond its thermal behavior, we synthesized a series of aripiprazole fatty acid prodrugs with different alkyl chain lengths (Figure 1). Through comprehensive characterization of crystal structures, physicochemical properties, and in vivo pharmacokinetic profiles in rats, we established the chain length–property correlation for the APZ prodrugs. Additionally, the comparisons with literature data from other prodrug systems may help clarify system-dependent variations, thereby providing a reference for the rational design of fatty acid prodrugs for long-acting injectable aqueous suspensions.

## 2. Materials and Methods

Aripiprazole (APZ) was obtained from Jiangsu Aikang Biopharmaceutical R&D Co., Ltd., Nanjing, China. Lauric acid (C12), palmitic acid (C16), arachidic acid (C20), dicyclohexylcarbodiimide (DCC), and carbamazepine were purchased from Aladdin Biochemical Technology Co., Ltd., Shanghai, China. 4-Dimethylaminopyridine (DMAP) and deuterated chloroform (CDCl_3_) were purchased from Maikelin Biotechnology Co., Ltd., Shanghai, China. Paraformaldehyde, Tween 20, toluene (Tol), isopropanol (IPA), dimethyl sulfoxide (DMSO), ethyl acetate (EtOAc), *n*-hexane, triethylamine (TEA), ammonia, sodium citrate, sodium dihydrogen phosphate dihydrate, and disodium hydrogen phosphate dodecahydrate were obtained from Nanjing Chemical Reagent Co., Ltd., Nanjing, China. Acetonitrile (HPLC grade) was purchased from Tedia Inc., Fairfield, OH, USA. 1,8-Diazabicyclo[5.4.0]undec-7-ene (DBU) was purchased from Bide Pharmatech Co., Ltd., Shanghai, China. Dichloromethane (DCM), ethanol (EtOH), and methanol (MeOH) were purchased from Titan Pharmatech Co., Ltd., Shanghai, China. All reagents and materials were used as received without further purification.

### 2.1. Preparation of APZ Form III and Synthesis of APZ-Fatty Acid Prodrugs

APZ Form III was prepared by refluxing 1 g of aripiprazole raw material in 10 mL of the mixed solvent of ethanol and water (75:25, *v*/*v*) at 75 °C for 2 h. Slow cooling to room temperature produced a white precipitate, which was collected by filtration and dried under vacuum at 40 °C for 3 days to yield aripiprazole monohydrate, which was then heated on a hot stage at 110 °C for 2 h to produce aripiprazole Form III. Unless otherwise specified, APZ described in this work refers to aripiprazole Form III.

APZ-fatty acid prodrugs (APZP12, APZP16, APZP20) were synthesized following a reported methodology with minor modifications (see Supplementary Materials) [22]. Characterization was conducted by ^1^H-NMR on a Bruker AVANCE 500 MHz spectrometer (Rheinstetten, Germany) using CDCl_3_ and by mass spectrometry on an Agilent Q-TOF 6545 instrument (Chicopee, MA, USA) operating in positive ion electrospray mode.

### 2.2. Preparation of APZ-Fatty Acid Prodrug Suspensions

APZ prodrugs were dispersed in an aqueous solution containing Tween 20 and Span 20. The suspensions were transferred into a zirconium oxide grinding chamber containing 0.8 mm zirconium oxide beads and subjected to wet milling in a planetary ball mill (Changsha Tianchuang Instrument Co., Ltd., Changsha, China). The pH of the obtained suspensions was adjusted to 7.0 by the addition of sodium phosphate dibasic anhydrous, sodium phosphate monobasic, and sodium hydroxide solution. A summary of the suspension formulation and processing parameters is available in Table S1.

### 2.3. Characterization of APZ-Fatty Acid Prodrugs and Suspensions

Single crystals of APZ-fatty acid prodrugs were grown by solvent evaporation. Briefly, APZ-fatty acid prodrug powder (20 mg) was dissolved in 6 mL of solvent (ACN for APZP12, IPA for APZP16, EtOH for APZP20). Each solution was subjected to membrane filtration (0.45 μm) to remove particulates. The clear filtrates were left at room temperature for gradual solvent evaporation, yielding single crystals suitable for analysis. Single-crystal X-ray diffraction (SCXRD) was conducted on a Bruker D8 Venture ECO instrument (Karlsruhe, Germany). The SAINT software 5.0 was employed for data integration, and the SADABS program was used for the empirical absorption correction of data. SHELXL-2014 was employed for structure solution and refinement via direct methods and the least-square method successively. The hydrogen atoms were refined by isotropic calculation.

Differential scanning calorimetry (DSC) was conducted on a TA Instruments Q2000 (New Castle, DE, USA) calibrated with indium. Samples weighing 3–5 mg were sealed in aluminum pans and heated to a certain temperature at 10 °C/min under a nitrogen flow of 50 mL/min. The thermograms were analyzed using TA Universal Analysis software 2000.

Thermogravimetric analysis (TGA) was performed on a TA Instruments Q500 (New Castle, DE, USA) with a platinum pan. Each sample (5–15 mg) was heated from ambient temperature to 500 °C at a ramp rate of 20 °C/min under a nitrogen purge of 40 mL/min.

Powder X-ray diffraction (PXRD) was carried out on a Rigaku SmartLab SE diffractometer (Tokyo, Japan) with a Cu Kα radiation (λ = 1.54178 Å). Each sample was placed on a glass plate and measured over a 2θ range of 3–40° using 0.02° steps at 40 kV and 40 mA.

Particle size distributions of the APZ-fatty acid prodrug suspensions were measured on a BT-9300 laser diffraction analyzer (Dandong Bettersize Instrument Co., Ltd., Dandong, China), and the *D*10, *D*50, and *D*90 values were recorded. The span value, indicating the distribution width, was calculated using the following relationship: (*D*90–*D*10)/*D*50.

The morphology of the APZ-fatty acid prodrug suspensions was characterized by scanning electron microscopy using a FEI Quanta FEG 250 instrument (Hillsboro, OR, USA). Imaging was performed at 20 kV with a working distance of 8.0–9.7 mm. Prior to examination, sample surfaces were sputtered with gold-palladium for conductivity.

### 2.4. Hirshfeld Surfaces and Crystal Voids Analysis of APZ-Fatty Acid Prodrugs

Intermolecular interactions of the prodrugs were characterized by Hirshfeld surfaces and two-dimensional (2D) fingerprint plots generated with CrystalExplorer software 17, offering a visual and quantitative assessment of intermolecular contacts across the series of alkyl chain lengths. The surfaces were color-mapped according to the normalized distance (d_norm_), where red, white, and blue regions denote strong, moderate, and weak intermolecular contacts, respectively. The 2D fingerprint plots provided a quantitative breakdown of these contacts. Crystal voids were calculated from procrystal electron density isosurfaces with a cutoff of 0.002 atomic units (a.u.) to quantify the unoccupied space within the unit cell.

### 2.5. Solubility and Lipophilicity of APZ-Fatty Acid Prodrugs

The solubility of APZ prodrugs was determined in a 1:1 (*v*/*v*) EtOH/acetate buffer (pH 5.0). An excess of powder was added to the medium, and the suspension was shaken at 37 °C for 24 h, followed by centrifugation at 13,000 rpm for 5 min. The resulting supernatant was diluted and analyzed by HPLC.

The lipophilicity of APZ-fatty acid prodrugs was assessed on a Shimadzu LC-20AT HPLC system with an Agilent Zorbax SB-C18 column (4.6 × 250 mm, 5 µm) kept at 40 °C. An isocratic mobile phase of acetonitrile and deionized water with 0.1% formic acid and 0.1% triethylamine (80:20, *v*/*v*) was pumped at 1.0 mL/min with UV detection at 254 nm. The capacity factor (k’) was derived from (*t*_R_ − *t*_0_)/*t*_0_, with *t*_R_ and *t*_0_ being the retention times of the APZ prodrugs and unretained solvent, respectively.

### 2.6. Wettability of APZ-Fatty Acid Prodrugs

For contact angle measurement, 100 mg of the sieved prodrug powder (80-mesh) was compressed into tablets with a hydraulic press under a load of 200 kg for 5 min to obtain a smooth surface. Surface wettability of the compacted tablets was then assessed by the sessile drop method on an OCA 20 instrument (DataPhysics Instruments GmbH, Filderstadt, Germany). Droplets of water containing 1 wt.% Tween 20 were dispensed onto the compacted tablet surface, with their droplet profiles monitored by a CCD camera and analyzed using the tangent method with the integrated software.

### 2.7. Plasma Cleavage Kinetics of APZ-Fatty Acid Prodrugs

Each prodrug was dissolved in methanol (100 μg/mL), and 10 μL aliquots were added to 50 μL of rat plasma. The mixtures were incubated at 37 °C for 2 h in triplicate. The sample were withdrawn and precipitated with 240 μL of acetonitrile at 5, 10, 15, 20, 30, 45, 60, 90, and 120 min. After mixing and centrifugation at 13,000 rpm for 5 min, the supernatant was analyzed by HPLC. Concentrations of APZ and its fatty-acid prodrugs were determined under different chromatographic conditions.

APZ-fatty acid prodrugs were quantified as described in Section 2.5 with minor modifications (Table S2). APZ concentrations were measured at 40 °C on an Agilent Zorbax SB-C18 column (4.6 × 250 mm, 5 µm) with UV detection at 254 nm. The mobile phase comprised acetonitrile and deionized water containing 0.1% (*v*/*v*) formic acid and 0.1% (*v*/*v*) ammonia (60:40, *v*/*v*), delivered at 1.0 mL/min.

### 2.8. Solid-State Stability of APZ-Fatty Acid Prodrugs

Each APZ prodrug powder was sieved through a 60-mesh sieve, and 30 mg samples were weighed into separate glass vials for each condition and time point. At each predetermined time point (0, 5, and 10 days), three individual vials (each containing 30 mg) for each prodrug were withdrawn for DSC, PXRD, and HPLC analysis. The experimental conditions included high temperature (60 °C), high humidity (92.5% RH), and strong light (4500 lux).

### 2.9. Stability of APZ-Fatty Acid Prodrug Suspensions

The APZ prodrug suspensions were stored at 4 °C for up to 28 days. At predetermined time intervals (0, 14, and 28 days), samples were withdrawn and analyzed for particle size (D50) and distribution (Span values) according to the method described in Section 2.3. All measurements were performed in triplicate.

### 2.10. In Vitro Release of APZ-Fatty Acid Prodrug Suspensions

Drug release from the APZ prodrug suspensions was performed on a USP Apparatus II (FADT-801 RC, FOCS Analysis Instrument Co., Ltd., Shanghai, China) at a paddle speed of 75 rpm. Suspensions were directly added into a dissolution medium comprising equal volumes of EtOH and acetate buffer (pH 5.0) maintained at 37 °C. At predetermined timepoints (5, 15, 30, 45, 60, 90, 120, 180, 240 min), 2 mL aliquots were withdrawn, immediately centrifuged at 13,000 rpm for 5 min, and the supernatant was quantified by HPLC.

### 2.11. Cytotoxicity of APZ-Fatty Acid Prodrug Suspensions

The cytotoxicity of APZ-fatty acid prodrug suspensions was evaluated using the CCK-8 assay in RAW 264.7 murine macrophages, acquired from the Chinese Academy of Sciences (Shanghai, China). The cells were cultured in high-glucose Dulbecco’s modified Eagle medium (DMEM) supplemented with 10% (*v*/*v*) fetal bovine serum (FBS), 1% penicillin, and 1% (*w*/*v*) streptomycin at 37 °C in a 5% CO_2_ atmosphere. The cells were plated at a density of 5 × 10^3^ cells per well in 96-well plates and incubated for 24 h. The culture medium was then replaced with 100 μL of fresh culture medium containing APZ-fatty acid prodrug suspensions at final APZ concentrations of 4–250 μM (*n* = 6). After a further 24-h exposure period, the cells were rinsed with phosphate-buffered saline (PBS) and incubated for 30 min with 10 μL of CCK-8 reagent diluted in 100 μL of fresh culture medium. Absorbance was recorded at 450 nm using an Infinite F50 microplate reader (Tecan). Cell viability was calculated using the formula ($A_{t}-A_{b}$)/($A_{c}-A_{b}$), where $A_{t}$, $A_{b}$, and $A_{c}$ represent the absorbances of the treated wells, the blank medium, and the untreated control cells, respectively.

### 2.12. Cell Uptake of APZ-Fatty Acid Prodrug Suspensions

For cellular uptake assessment, RAW 264.7 cells were plated at a density of 1.0 × 10^5^ cells per well in 12-well plates and incubated for 24 h. The cells were then incubated with fresh culture medium containing APZ prodrug suspensions (final APZ concentration of 8 μM) for 1, 2, 4, and 8 h. After treatment, cells were rinsed with PBS at room temperature three times to remove extracellular prodrugs, followed by centrifugation at 2000 rpm for 5 min. The pellets were resuspended in acetonitrile and sonicated at room temperature to release intracellular drugs. After centrifugation, the supernatant was directly analyzed by LC-MS/MS for the quantification of intracellular concentrations of APZ and APZ prodrugs without solvent evaporation.

### 2.13. Pharmacokinetics of APZ-Fatty Acid Prodrug Suspensions

#### 2.13.1. Animals

Experimental animals were SPF-grade male Sprague-Dawley (SD) rats (220–250 g), purchased from Vital River Co., Ltd., Beijing, China. Animals were housed in a standard procedure room within the SPF-grade animal facility under controlled environmental conditions (24 ± 2 °C, 50 ± 20% humidity, 12 h light/dark cycle, below 60 decibels) with ad libitum access to food and water. The animal experiments reported in this study were conducted in accordance with the guidelines of the Institutional Laboratory Animal Management Committee of China Pharmaceutical University and approved by the Animal Ethics and Welfare Committee of the university (Approval No. YSL-202504060; Approval date: 14 April 2025).

Sprague-Dawley rats, a widely used pharmacokinetic model, were chosen for their suitability for repeated blood sampling and intramuscular injection, as well as the availability of extensive reference data. Although species differences preclude direct extrapolation to humans, the observed chain-length trends remain informative.

#### 2.13.2. Experimental Design

Eighteen male SD rats were randomly divided into three groups (*n* = 6 per group) using a computer-generated randomization sequence (Excel RAND 5function). Following a 7-day acclimatization period, each group received a single intramuscular injection of the APZP12, APZP16, and APZP20 suspension, corresponding to 42 mg/kg of APZ. Approximately 0.5 mL of blood was withdrawn from the retro-orbital venous plexus and transferred into heparin-containing tubes at 0, 2, 4, and 8 h, and on days 1, 2, 3, 5, 7, 10, 14, 19, 25, 30, and 35 after administration. Plasma was isolated by centrifugation at 4000 rpm for 15 min, and was collected from the supernatants and stored at −80 °C until quantitative analysis. All blood collections were performed by trained technicians using a gentle technique to minimize discomfort to the animals.

#### 2.13.3. Sample Analysis

A 90 μL aliquot of plasma was spiked with 10 μL of the internal standard solution (2 μg/mL) and vortexed for 1 min. Then, 400 μL of acetonitrile was added to protein precipitation. The mixture was vortexed for 5 min and subsequently centrifuged at 13,000 rpm and 4 °C for 15 min. The supernatant was carefully collected and injected into the LC-MS/MS system for the analysis.

Plasma APZ levels were quantified using a Shimadzu LCMS-8045 liquid chromatography-tandem mass spectrometry (LC-MS/MS, Tokyo, Japan). Chromatographic separation was achieved using a Waters C18 column (4.6 × 150 mm, 5 µm), operated at 38 °C. An isocratic mixture of acetonitrile and purified water (85:15 *v*/*v*) containing 0.05% formic acid and 0.05% ammonium hydroxide was delivered at a flow rate of 0.5 mL/min. Multiple reaction monitoring mode was used for detection, with the ion transitions set at *m*/*z* 448.3 → 285.2 for APZ and *m*/*z* 237.2 → 194.2 for carbamazepine (the internal standard).

The method was validated over a linear range of 0.1–200 ng/mL for all biological matrices, with excellent correlation (R^2^ > 0.999) achieved by weighted least squares regression (1/x^2^).

#### 2.13.4. Data Analysis

A noncompartmental analysis was performed to obtain pharmacokinetic parameters using DAS software 2.0 (Chinese Pharmacological Society, China). Statistical analysis was conducted using GraphPad Prism software 10.1.2. Pairwise comparisons between groups were performed using the Student’s *t*-test, with *p* < 0.05 considered statistically significant, and all values expressed as mean ± SD.

#### 2.13.5. Histomorphology

At the designated sampling intervals after intramuscular injection of the APZ prodrug suspensions, the rats were sacrificed and the tissue surrounding the administration site was collected. Samples were immersed in 4% paraformaldehyde (G1101, Wuhan Servicebio Technology Co., Ltd., Wuhan, China)) for no less than 48 h, followed by routine paraffin processing, including sequential ethanol dehydration and xylene clearing. The paraffin blocks were cut into 4 μm sections. For histological assessment, sections were subjected to hematoxylin and eosin (H&E) staining, whereas Masson’s trichrome staining was used to assess collagen deposition. Stained sections were examined and imaged using a Nikon Eclipse E100 optical microscope (Tokyo, Japan) fitted with a digital imaging system. The acquired histological images were processed using CaseViewer 2.4 software.

## 3. Results

### 3.1. Synthesis and Solid-State Characterization of APZ-Fatty Acid Prodrugs

**Figure 2:**
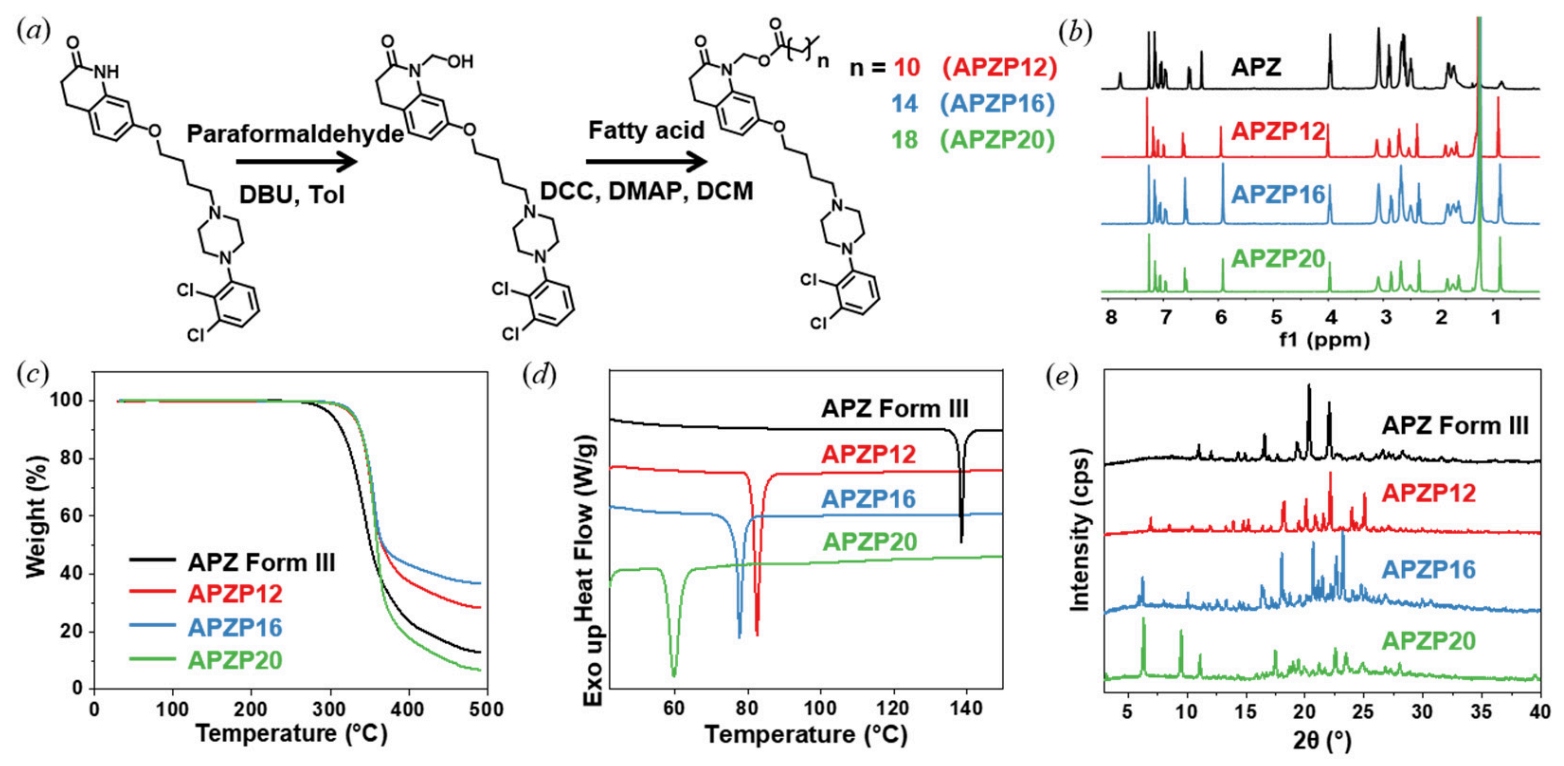
Synthesis and solid-state characterization of APZ-fatty acid prodrugs. (**a**) Schematic representation of the synthetic route, (**b**) ^1^H-NMR spectra, (**c**) TGA thermograms, (**d**) DSC thermograms, and (**e**) PXRD patterns of APZ-fatty acid prodrugs.

Three APZ prodrugs (APZP12, APZP16, APZP20) were synthesized via a two-step procedure (Figure 2a). Initially, APZ was subjected to hydroxymethylation to yield the intermediate compound hydroxymethyl APZ (HAPZ). Subsequently, HAPZ underwent esterification with saturated fatty acids of different chain lengths (C12, C16, and C20) to yield the corresponding prodrugs. The successful conjugation of the fatty acid to APZ was confirmed by ^1^H-NMR spectroscopy. Specifically, the ^1^H-NMR spectra exhibited a triplet signal at 5.92–5.95 ppm, assignable to the methylene protons of the *-N-CH2-OCO-* linker between APZ and the fatty acid, and a multiplet signal at 1.24–1.38 ppm, corresponding to the methylene groups (*-CH_2_-*) of the fatty acid chains (Figure 2b). These assignments provided direct evidence for the substitution of the original amide group in APZ [23]. Mass spectrometry further supported the conjugation, with the observed molecular ion peaks for APZP12, APZP16, and APZP20 being in good agreement with their respective theoretical values (Supplementary Materials).

The solid-state characterization of the three APZ prodrugs was performed using TGA, DSC, and PXRD. As the commercially employed crystal form in oral formulations, APZ Form III was prepared and characterized as a reference [24]. The TGA results (Figure 2c) indicated no apparent weight loss below 150 °C, confirming the anhydrous nature of the APZ prodrugs. Furthermore, the prodrugs exhibited superior thermal stability, with decomposition temperatures exceeding 300 °C and minimal variation among the three prodrugs. DSC thermograms (Figure 2d) displayed a single, sharp endothermic melting peak for each prodrug, indicative of high phase purity. The melting points were determined to be 80.60 ± 0.61 °C (APZP12), 76.72 ± 0.83 °C (APZP16), and 56.36 ± 0.48 °C (APZP20), all markedly lower than that of APZ Form III (139 °C) [25]. A notable inverse relationship between the alkyl chain length and melting temperature was observed. Comparative PXRD analysis (Figure 2e) revealed distinct variations in diffraction peak positions between the prodrugs and APZ Form III, reflecting structural differences arising from variations in molecular conformation, intermolecular interactions, and packing arrangements.

### 3.2. Crystal Structure Analysis of APZ-Fatty Acid Prodrugs

Plate-like crystals of the APZ prodrugs were cultivated via the slow solvent evaporation method and subsequently analyzed by single-crystal X-ray diffraction. Notably, the single crystals of APZP20 exhibited weak diffraction at high angles and poor overall data quality, preventing structure solution, likely as a result of the conformational flexibility of the long-chain fatty acid chains. Both APZP12 and APZP16 crystallized in the monoclinic *P*2_1_/c space group, with each unit cell containing four prodrug molecules. Other crystallographic parameters are summarized in Table S3. The simulated PXRD patterns derived from the single-crystal structural data matched well with the experimental patterns, confirming the phase purity of the bulk prodrug samples (Figure S1).

**Figure 3:**
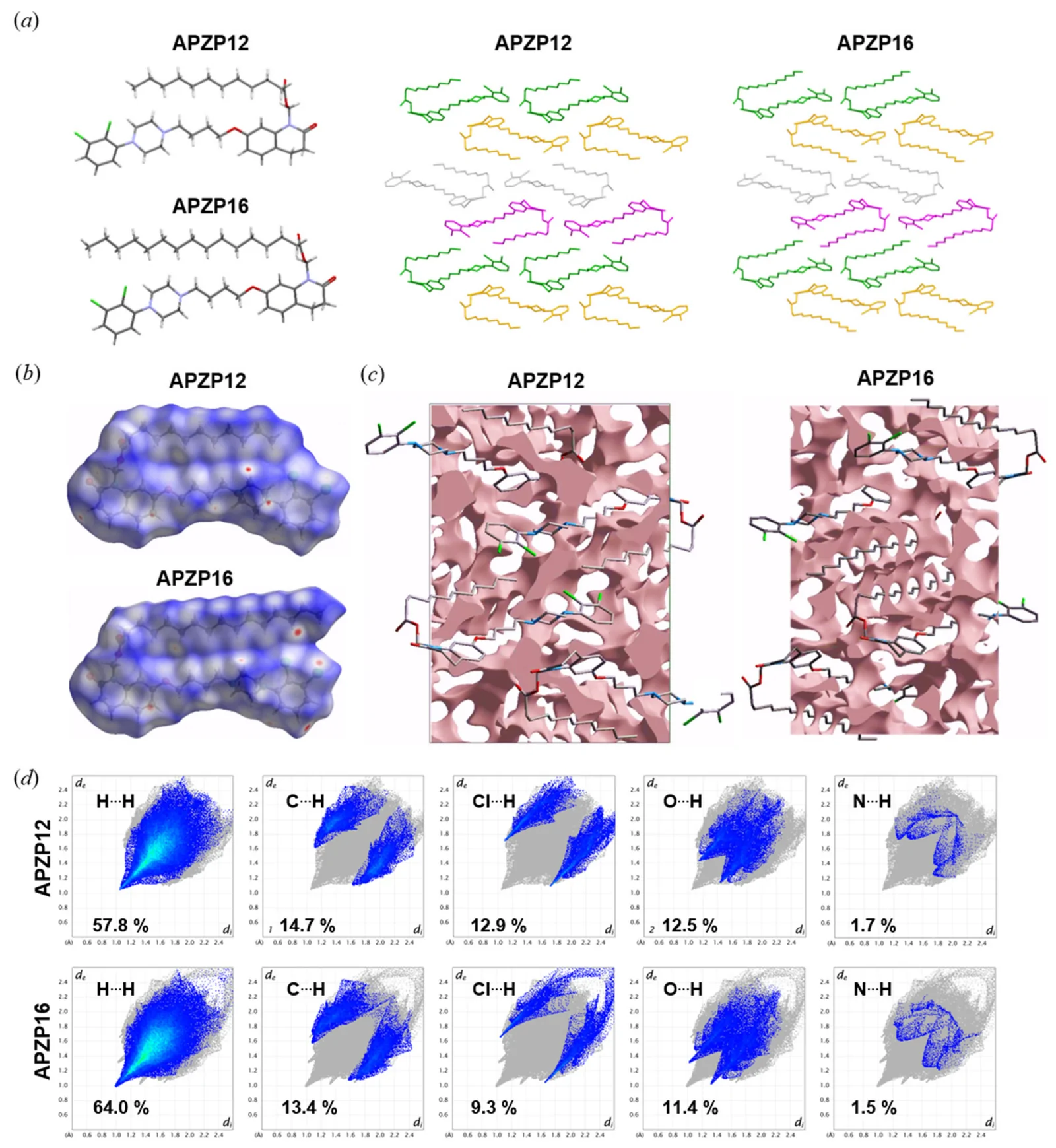
Crystal structure analysis of APZ-fatty acid prodrugs. (**a**) Molecular conformation and packing, (**b**) Hirshfeld surface, with red, white, and blue regions representing strong, moderate, and weak intermolecular contacts, respectively, (**c**) crystal void maps, with pink regions indicating the void spaces within the unit cell, (**d**) 2D fingerprint plots as a function of the *d*_e_ and *d*_i_ distances of prodrugs. Disordered alkyl chain segments have been omitted for clarity.

The two APZ prodrugs exhibited isostructural packing (Figure 3). Each prodrug molecule adopted a U-shaped conformation with the aripiprazole moiety and the fatty acid chain aligned parallel to each other (Figure 3a); further details regarding the crystal packing are provided in Figures S2 and S3. In these prodrugs, intermolecular hydrogen bonds and π–π interactions were found to be extremely weak (Tables S4 and S5); instead, multiple prodrug molecules were assembled via short contacts between carbonyl oxygen atoms and aromatic hydrogen atoms, extending along the *c*-axis into a zigzag chain (Figure 3). Hirshfeld surface analysis of the two APZ prodrugs revealed comparable distributions of color-coded regions (Figure 3b), suggesting similarity in their interaction sites [26,27]. Owing to its longer fatty acid chain, APZP16 exhibited a higher proportion of H···H contacts and consequently a lower proportion of polar contacts, indicating potentially slower solvation (Figure 3d) [28]. In addition, the unoccupied space within the prodrug lattice was mapped and quantitatively calculated to understand the packing efficiency and structural compactness of the supramolecular architecture (Figure 3c). APZP12 possessed a total void volume of 489.83 Å^3^, accounting for 13.6% of the total unit cell volume (3587.8 Å^3^), whereas APZP16 possessed a total void volume of 499.37 Å^3^ corresponding to 12.7% of the total unit cell volume (3924.9 Å^3^). This relatively low void volume in APZP16 suggests more efficient molecular packing, with constituent molecules more densely arranged with minimal interstitial gaps [29].

### 3.3. Physicochemical Properties and Hydrolysis Kinetics of APZ-Fatty Acid Prodrugs

**Figure 4:**
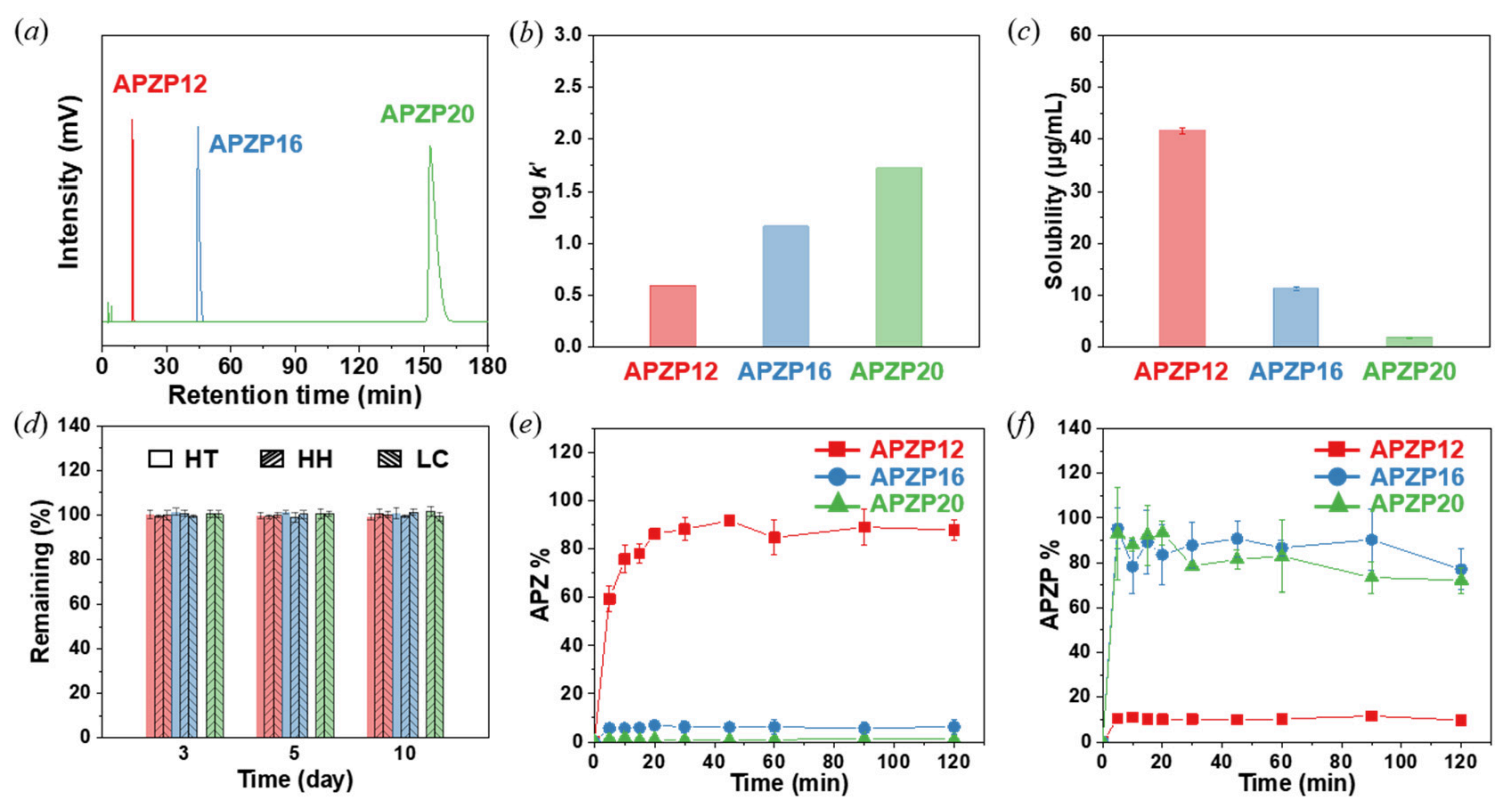
Physicochemical properties and hydrolysis kinetics of APZ-fatty acid prodrugs. (**a**) Chromatographic peaks of the prodrugs, (**b**) log k’ values calculated from retention times using log k’ = log(*t*_R_−*t*_0_)/*t*_0_, (**c**) solubility in a 1:1 (*v*/*v*) mixture of EtOH and acetate buffer (pH 5.0) at 37 °C, (**d**) stability under 60 °C (high temperature, HT), 92.5% relative humidity (high humidity, HH), and 4500 lux (light condition, LC), with red, blue, and green bars representing APZP12, APZP16, and APZP20, respectively, (**e**,**f**) hydrolysis kinetics of APZ prodrugs in rat plasma over 2 h.

The physicochemical properties of fatty-acid prodrugs (e.g., solubility, lipophilicity, stability) critically affect the final formulation quality, stability, and in vivo performance [2,12,30]. As the chain length increased, the lipophilicity of the prodrug increased progressively, as assessed by HPLC based on the well-established correlation between the logarithm of the capacity factor (log k’) and lipophilicity [31,32]. The chromatograms and retention times are provided in Figure 4a and Table S6; both the retention times and capacity factors (k’) of the prodrug exhibited a progressive increase with longer alkyl chain lengths (Figure 4b). An opposite trend was observed for solubility, which was assessed in a 1:1 (*v*/*v*) mixture of EtOH and acetate buffer (pH 5.0). The acetate buffer component was chosen based on the FDA Dissolution Methods database recommendation for aripiprazole extended-release suspensions, while ethanol was incorporated to ensure that the solubility levels of all prodrugs fell within a quantifiable range for a reliable comparison. The solubility decreased progressively with increasing alkyl chain length in the order of APZP12 (41.53 ± 0.59 μg/mL), APZP16 (11.19 ± 0.36 μg/mL), APZP20 (1.83 ± 0.14 μg/mL) (Figure 4c). Additionally, APZP12 and APZP16 exhibited superior stability under accelerated conditions (60 °C, 92.5% relative humidity, 4500 lux), whereas APZP20 underwent severe degradation under 60 °C, with its HPLC signal becoming undetectable, accompanied by noticeable changes in its DSC and PXRD patterns (Figure 4d, Figures S4 and S5). The physicochemical properties of the APZ prodrugs are summarized in Table 1.

The conversion of pharmacologically inactive prodrugs into their active parent drugs relies on enzymatic or chemical hydrolysis under physiological conditions. Therefore, the hydrolysis kinetics of the APZ prodrugs in rat plasma were evaluated by quantifying the concentrations of both APZ and the corresponding prodrugs at predetermined time points (Figure 4e,f). The results demonstrated an inverse relationship between hydrolysis kinetics and alkyl chain length. Specifically, prodrugs with longer alkyl chains (APZP16, APZP20) exhibited comparatively higher stability, releasing less than 20% of APZ within 2 h (Figure 4e). Conversely, APZP12 underwent rapid enzymatic cleavage, with over 50% of APZ released within 5 min. These trends were further supported by the prodrug concentration-time profiles, which revealed high prodrug retention for APZP16 and APZP20 (>70%), in contrast to the low retention observed for APZP12 (<20%) (Figure 4e,f). This result is likely associated with the reduced steric hindrance for APZP12, which facilitates the accessibility of the ester bond to lipase [33], while the contribution of thermotropic behavior appears to be relatively minor in this context, given that APZP12 exhibits the highest melting point, corresponding to the greatest lattice energy among the three prodrugs.

### 3.4. Characterization and Evaluation of APZ-Fatty Acid Prodrug Suspensions

**Figure 5:**
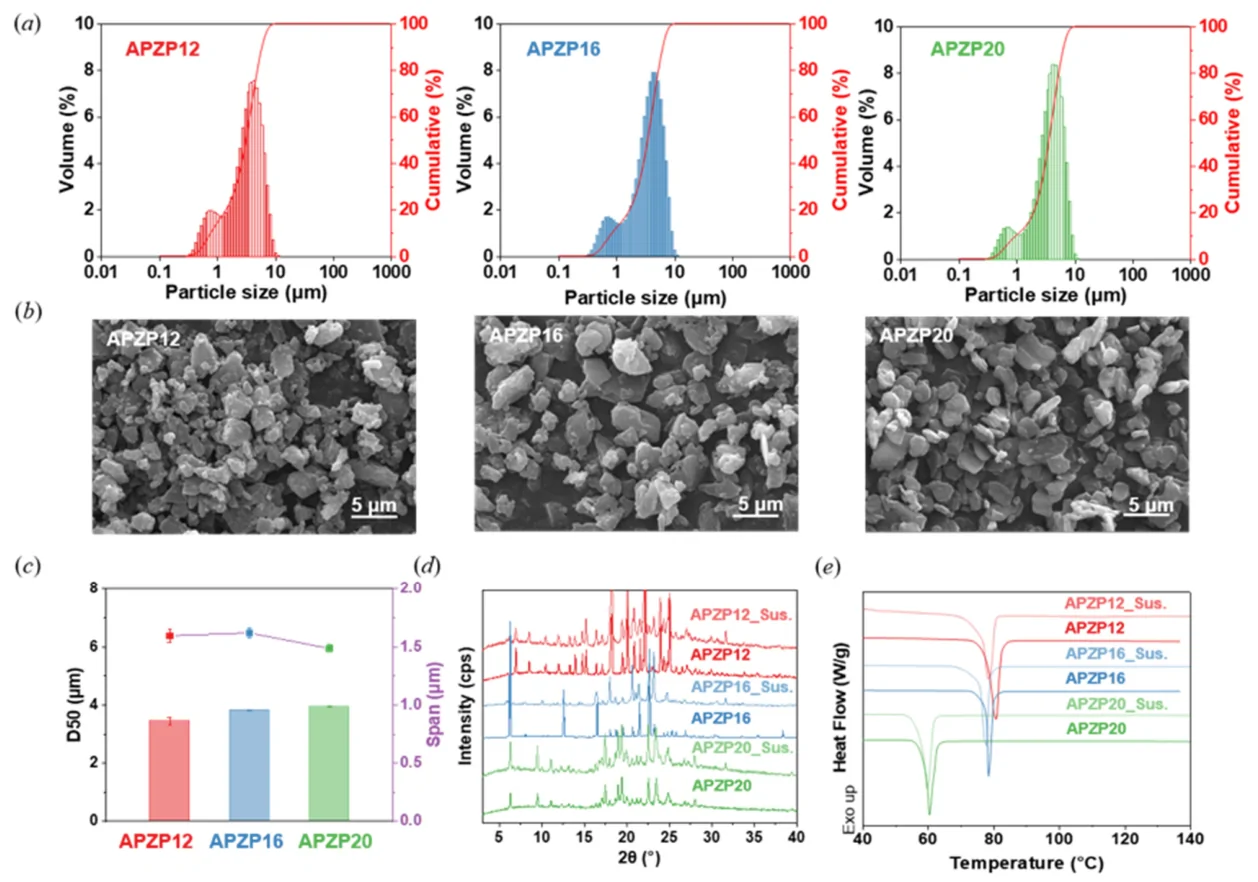
Characterization of APZ-fatty acid prodrug suspensions. (**a**) Particle size distributions profiles, with the red line representing the cumulative distribution curves, (**b**) SEM images, (**c**) D50 and Span values, (**d**) PXRD patterns, and (**e**) DSC thermograms of the APZ-fatty acid prodrug suspensions.

To examine the impact of alkyl chain length on biopharmaceutical performance, three APZ prodrug suspensions were prepared via wet milling. Top-down approach is an established and scalable technique that has been successfully employed in the commercial manufacturing of several approved injectable suspensions [4,5]. Importantly, wet milling achieves effective particle size reduction in these poorly soluble prodrugs without the use of organic solvents, offering an effective and industrially viable formulation strategy. The formulation and processing parameters (Table S1) were kept comparable across all suspensions, with the alkyl chain length of the prodrug being the sole variable. The bulk prodrug powders exhibited distinct morphologies prior to milling (Figure S6), whereas after wet milling, all three suspensions exhibited similar particle size distributions (Figure 5a) and morphologies (Figure 5b), consisting of uniformly sized, well-defined particles with a median diameter of approximately 3.8 μm (Figure 5c). Subsequently, PXRD and DSC characterizations were performed to assess the stability of the APZ prodrugs during the wet-milling process. Close agreement was observed between the diffraction patterns obtained before and after processing, indicating preservation of molecular bonding and packing characteristics throughout the formulation development (Figure 5d). Meanwhile, DSC thermograms revealed a slight depression in the melting temperatures of the lyophilized powders derived from the prodrug suspensions compared to the original prodrugs, which can be likely ascribed to the particle size reduction (Figure 5e) [34]. The APZ prodrug suspensions exhibited good physical stability at 4 °C, as evidenced by stable particle size and Span values (Figure S7), which are commonly used as direct indicators of stability for suspensions, although zeta potential was not measured.

**Figure 6:**
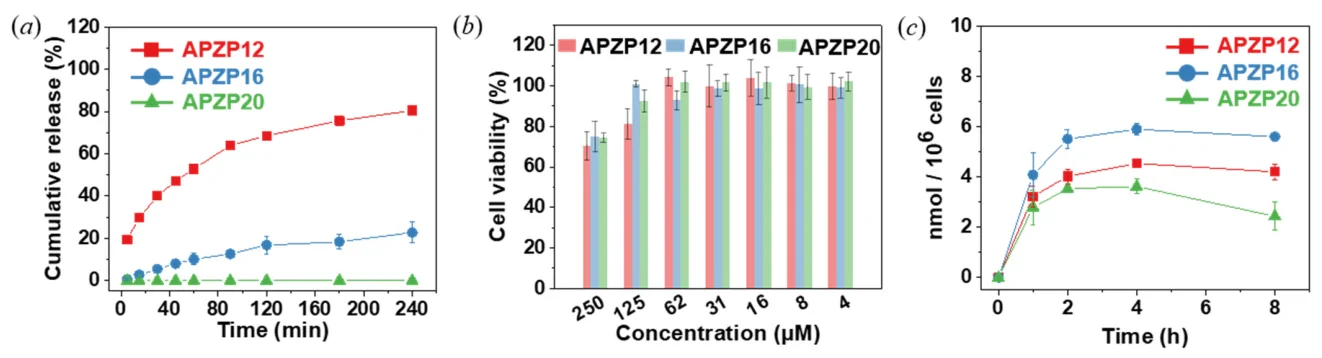
Evaluation of APZ-fatty acid prodrug suspensions. (**a**) In vitro drug release profiles, (**b**) cytotoxicity in RAW 264.7 cells, and (**c**) cellular uptake of the APZ prodrug suspensions in RAW 264.7 cells.

The in vitro release behavior of the APZ prodrug suspensions was examined under sink conditions (Figure 6a). APZP12 suspension exhibited the fastest release, with approximately 50% of the prodrug released within the first hour. In contrast, the release of APZP16 and APZP20 was substantially slower, with less than 20% of the prodrug released over the same period. This chain length-dependent release behavior corroborated differences in wettability and polar contact abundance among the APZ prodrugs: shorter alkyl chains possess better wettability and a high proportion of polar contacts (Figure S8), which likely facilitates solvation, thereby leading to an accelerated release.

The cytotoxicity of the APZ prodrug suspensions was further examined in RAW 264.7 macrophages with the CCK-8 assay at concentrations ranging from 4 to 250 μM (Figure 6b). All suspensions induced a dose-dependent reduction in cell viability, with cytotoxicity increasing at higher prodrug concentrations. No significant differences in cytotoxicity were observed among APZP12, APZP16, and APZP20 at equivalent concentrations. Cell viability remained above 80% at concentrations below 62 μM, indicating negligible cytotoxicity, whereas a marked decrease in cell viability occurred at 125 and 250 μM.

Given that macrophage uptake can prolong half-life by facilitating cellular and tissue drug retention, formulation uptake was assessed in RAW 264.7 cells after treatment with APZP12, APZP16, APZP20 over an 8 h period (Figure 6c) [35,36]. All three prodrugs exhibited an initial increase in uptake followed by a plateau, likely reflecting saturation of the cellular uptake capacity [37]. At 8 h, intracellular prodrug levels were 4.20, 5.60, and 2.43 nmol/million cells for APZP12, APZP16, and APZP20, respectively. Notably, APZP16, featuring an intermediate chain length, exhibited significantly higher intracellular prodrug levels than APZP12 and APZP20. This optimal performance is likely attributed to its favorable affinity to membrane components [38,39]. Additionally, APZ concentrations in the corresponding samples were determined to evaluate the intracellular conversion of the prodrug to its active parent drug. Consistent with the formulation release profiles, the intracellular APZ levels decreased progressively with increasing alkyl chain length (Figure S9).

### 3.5. Pharmacokinetic Study of APZ-Fatty Acid Prodrugs

**Figure 7:**
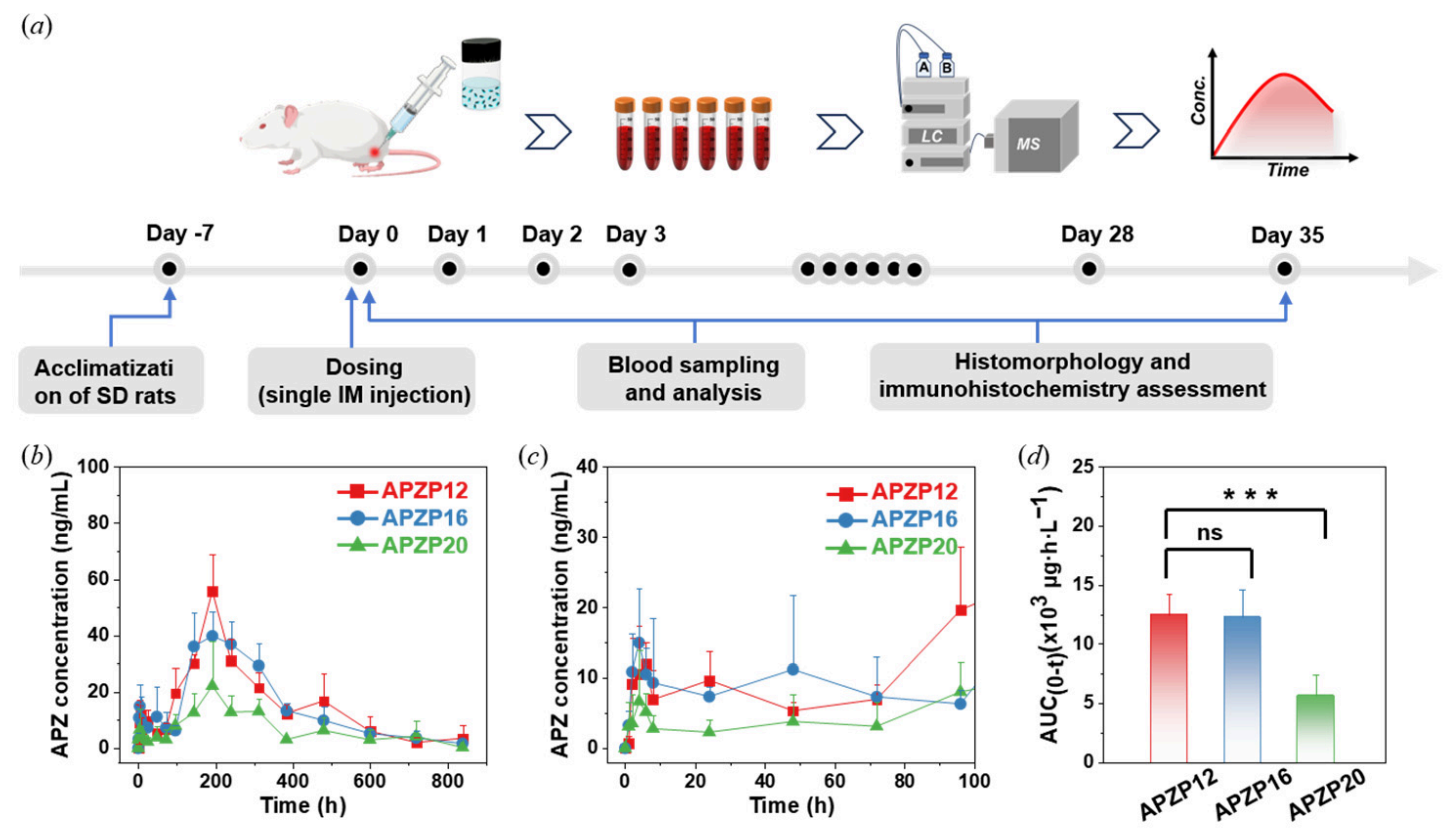
Pharmacokinetics of APZ prodrug suspensions after a single intramuscular injection to rats at a dose of 42 mg/kg (APZ equivalent). (**a**) Experimental schedule for the in vivo study, (**b**) mean plasma concentration–time curves of APZ over 0–840 h, (**c**) magnified mean plasma concentration–time curves of APZ over 0–100 h, (**d**) comparative pharmacokinetic parameters (*AUC*). Data are shown as mean ± standard deviation (*n* = 6). ***: *p* < 0.001, ns: not significant. Several elements in the image were obtained from BioGDP.com [21].

To examine whether the fatty acid chain length affects APZ prodrug performance in vivo, pharmacokinetic studies were performed in male SD rats following the study design outlined in Figure 7a. Due to the high esterase activity in rat blood, which rapidly hydrolyzes the prodrugs, plasma concentrations of the active moiety (APZ) were determined by LC-MS/MS rather than those of the parent prodrugs [40]. The resulting plasma APZ concentration–time profiles for APZP12, APZP16 and APZP20 suspensions are presented in Figure 7b,c. All suspensions sustained drug release for up to one month and exhibited a distinct double-peak plasma concentration–time profile following intramuscular injection. This phenomenon is known to result from the initial absorption of free molecules in solution together with the rapid dissolution of ultrafine particles (contributing to the first peak), succeeded by macrophage-mediated intracellular dissolution combined with continuous depot release (contributing to the second peak) [6,41,42]. Notably, the peak plasma concentrations of the three prodrugs followed the order APZP12 (52.4 ± 14.3 ng/mL) > APZP16 (44.4 ± 6.3 ng/mL) > APZP20 (25.9 ± 12.6 ng/mL), which was consistent with the order of their in vitro solubility. Pharmacokinetic parameters were subsequently derived and are provided in Table S7. The area under the concentration–time curve (*AUC*_0 − t_) of APZP12 (12,491.4 ± 1730.5 μg·h·L^−1^) and APZP16 (12,268.4 ± 2352.6 μg·h·L^−1^) was significantly higher than that of APZP20 (5658.2 ± 1818.1 μg·h·L^−1^), with no significant difference between APZP12 and APZP16, despite a minor numerical difference (Figure 7d).

### 3.6. Histological Evaluation of APZ-Fatty Acid Prodrugs

**Figure 8:**
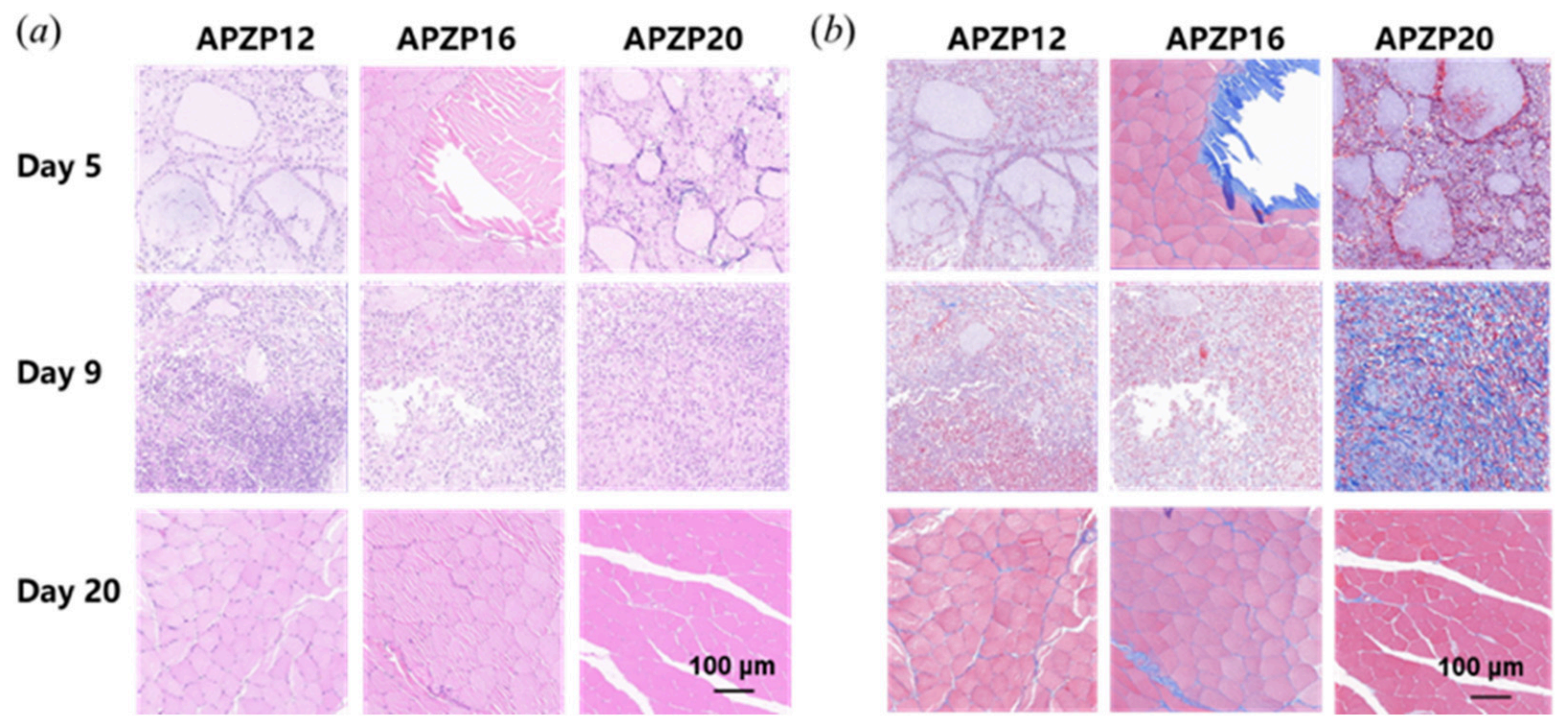
Histological analysis of rat muscle tissues at the injection sites. (**a**) HE-stained and (**b**) Masson-stained tissue sections. Scale bar: 100 μm. In the HE-stained sections, purple/blue indicates cell nuclei and pink indicates cytoplasm/muscle tissue. In the Masson-stained sections, blue/green indicates collagen fibers and red indicates muscle tissue.

The muscle tissues at the injection sites administered with the APZ prodrug suspensions were isolated, fixed, and histopathologically examined. Consistent with previous observations of long-acting injectable aqueous suspensions, all formulations triggered a localized inflammatory response, a typical manifestation of the normal foreign-body response associated with the clearance of a particulate depot [43,44,45]. On day 5 post-injection, loose, irregular cavities surrounded by collagen fibers and phagocytes were present (Figure 8), which arose from the washout of particle aggregates driven by the rapid diffusion of the suspension medium and subsequent muscle contraction [14]. By day 9, these cavities had markedly decreased in size, reflecting continuous depot dissolution and the consequent volume loss. In parallel, the number of inflammatory cells increased markedly compared with day 5, indicating a sustained immune response toward the residual depot. By day 20, after the depot had undergone nearly complete dissolution and absorption, the inflammatory response had largely resolved, and the local tissue architecture had uneventfully returned to its near-normal state. Altogether, these histopathological findings suggest that the inflammatory response caused by the injection and drug particles were transient and fully reversible, thereby demonstrating that the developed APZ prodrug suspensions provide a biocompatible and safe platform for long injectable applications.

## 4. Discussion

Fatty acid-prodrug based long-acting injectable aqueous suspensions have become a clinically established formulation platform. Nevertheless, the selection of an appropriate alkyl chain length for such prodrugs remains largely empirical, relying on extensive trial-and-error and thus incurring substantial R&D costs. In this study, three APZ prodrugs with different alkyl chain lengths were synthesized, and their physicochemical properties and pharmacokinetic behavior were evaluated as a function of chain length. With increasing alkyl chain length, the APZ prodrugs exhibited a progressive decline in melting points, aqueous solubility, wettability, and solid-state stability, which was accompanied by a concomitant elevation in flexibility, lipophilicity, and plasma stability.

Notably, such chain length–property correlations vary across different prodrug systems (Table S8) [15,33,35,46,47,48,49,50,51,52,53,54]. For example, in the paliperidone prodrug series, longer alkyl chains lead to higher melting points and lower solubility, whereas no such melting point dependence on chain length is observed for brexpiprazole prodrugs. To explore the structural origins driving these system-dependent correlation differences, we compared the available crystallographic data for each prodrug series (Figure S10). For the aripiprazole and paliperidone series, the molecular conformations are highly consistent with negligible differences in torsion angles; conversely, brexpiprazole prodrugs exhibit remarkable variations in both molecular conformation and torsion angles. These findings suggest that the preservation of isostructural crystal packing governs these predictable property trends in the fatty acid prodrugs, though further validation across a broader range of systems is warranted. When isostructural packing is maintained, the physicochemical properties evolve in a predictable trend with alkyl chain length. Conversely, in systems where chain elongation alters the molecular conformation or disrupts the crystal packing motif, the chain length–property correlation becomes highly unpredictable. Beyond the packing mode, the inherent flexibility of the parent drug may also influence the chain length-property correlation. For instance, the relatively rigid paliperidone, with only two known packing polymorphs, retains isostructural packing across a broad alkyl chain range (C4–C16) [54,55,56]. In contrast, the flexible APZ exhibits multiple conformational polymorphs, yielding discontinuous isostructural intervals [57]. This observation is consistent with literature reports of non-linear melting point trends across different chain lengths, suggesting that the isostructural packing observed in our study may represent only a specific chain length window, although this possibility warrants further validation [12]. These findings underscore the importance of solid-state study in developing fatty acid prodrug-based long-acting suspensions, and may offer a useful reference for evaluating chain length-dependent properties during chain modification.

Among the APZ prodrugs, APZP12 is the clinically established chain length, and the present findings rationalize this clinical preference. APZP12 exhibited the highest melting point among the three prodrugs, reflecting a higher lattice energy that is advantageous during the wet-milling process, as it minimizes the risk of shear-induced amorphous formation and subsequent Ostwald ripening [58,59,60]. Additionally, APZP12 exhibited relatively higher solubility and a faster hydrolysis rate in rat plasma, which may have contributed to its favorable PK profile, achieving therapeutic concentrations while mitigating the risk of prolonged in vivo retention. In contrast, the overly extended chain in APZP20 drastically compromised its solubility and plasma cleavage kinetics, which may have led to its insufficient systemic exposure. Notably, although APZP16 (intermediate chain length) demonstrated lower solubility and improved plasma stability in vitro, its bioavailability was comparable to that of APZP12, which was likely attributed to the compensatory effects of its higher cellular uptake on drug absorption. However, its lower plasma concentration might still pose a risk of a prolonged subtherapeutic phase, known as the PK tail [36]. Therefore, APZP12 achieves a favorable balance between physicochemical properties and pharmacokinetic performance, making it a suitable candidate for long-acting injectable formulation development.

## 5. Conclusions

In summary, this study investigated three aripiprazole fatty-acid prodrugs and revealed a clear chain length dependence in their physicochemical and biopharmaceutical properties. Among them, APZP12 exhibited the most favorable pharmacokinetic profile, which appears to result from a combination of higher aqueous solubility and faster hydrolysis kinetics, providing mechanistic insight beyond its relatively high melting point alone. In addition, cross-system comparisons of crystallographic data suggest that the preservation of isostructural packing enables the reliable prediction of chain length–property correlations across different fatty acid-prodrug systems. These findings highlight the critical role of isostructural packing and offer a useful reference for alkyl chain selection in the design of fatty acid-based prodrugs for long-acting injectable suspensions.

## Figures and Tables

**Table 1:** Physicochemical properties of APZ-fatty acid prodrugs.

	APZP12	APZP16	APZP20
Mw	660.69	716.80	772.94
Melting point (°C) ^a^	80.6 ± 0.6	76.7 ± 0.8	56.4 ± 0.5
log k’ ^b^	0.59	1.16	1.72
Solubility (μg/mL) ^a,c^	41.53 ± 0.59	11.19 ± 0.36	1.83 ± 0.14

^a^ Data are shown as mean ± SD (*n* = 3). ^b^ Determined by HPLC. ^c^ Measured in EtOH/acetate buffer (pH 5.0, 1:1, *v*/*v*).

## Data Availability

The original contributions presented in this study are included in the article or the Supplementary Materials. Further inquiries can be directed to the corresponding author.

## Supplementary Materials

The following supporting information can be downloaded at: https://www.mdpi.com/article/10.3390/pharmaceutics18080911/s1, S1. Synthesis of APZ fatty-acid prodrugs; Figure S1: Experimental (Exp.) and simulated (Sim.) PXRD patterns of APZ-fatty acid prodrugs; Figure S2: Crystal structure of APZP12. (a) Molecular conformation of prodrug, (b,c) molecular packing viewed along the *b*-axis (b) and the *a*-axis (c); Figure S3: Crystal structure of APZP16. (a) Molecular conformation of prodrug, (b,c) molecular packing viewed along the *b*-axis (b) and the *a*-axis (c); Figure S4: PXRD patterns of APZ-fatty acid prodrugs after 3, 5, and 10 days of storage under 60 °C, 92.5% RH, and 4500 lux light exposure; Figure S5: DSC thermogram of APZP20 after storage at 60 °C for 3 days; Figure S6: SEM images of APZ-fatty acid prodrugs; Figure S7: The D50 and Span value of APZ prodrug suspension vs. time under 4 °C; Figure S8: Contact angle of a water droplet containing 1 wt% Tween 20 on the surface of fatty acid prodrug tablets; Figure S9: Intracellular APZ levels after treatment with prodrug suspensions. Missing values indicate concentration levels below the limit of detection; Figure S10: Comparative crystallographic data for aripiprazole, paliperidone, and brexpiprazole fatty acid prodrugs. For the aripiprazole and paliperidone series, the molecular conformations are highly consistent with negligible differences in torsion angles, which may account for the correlation between chain length and prodrug properties. In contrast, brexpiprazole prodrugs exhibit remarkable variations in both molecular conformation and torsion angles, which might explain the absence of a clear chain length dependence in this system; Table S1: Process parameters for the milling of APZ-fatty acid prodrug suspensions; Table S2: HPLC parameters for the analysis of APZ-fatty acid prodrugs; Table S3: Crystallographic parameters of APZ-fatty acid prodrugs; Table S4: Hydrogen bond of APZ-fatty acid prodrugs; Table S5: Short ring-interactions with Cg-Cg distances < 6.0 A and Beta < 60° in APZ-fatty acid prodrugs; Table S6: Retention time and capacity factor of APZ fatty-acid prodrugs; Table S7: Pharmacokinetic parameters of APZ after intramuscular administration of three APZ-fatty acid prodrug suspensions to rats (mean ± standard deviation, *n* = 6); Table S8: Research on the relationship between fatty acid alkyl chain length and prodrug properties.

## Abbreviations

The following abbreviations are used in this manuscript:

ACN	Acetonitrile
AIDs	Acquired immunodeficiency syndrome
APZ	Aripiprazole
APZP12	Aripiprazole lauroxil
APZP16	Aripiprazole palmitate
APZP20	Aripiprazole arachidate
AUC	Area under the curve
C12	Lauric acid
C16	Palmitic acid
C20	Arachidic acid
CDCl_3_	Deuterated chloroform
DBU	1,8-Diazabicyclo[5.4.0]undec-7-ene
DCC	Dicyclohexylcarbodiimide
DCM	Dichloromethane
DMAP	Dimethylaminopyridine
DMEM	Dulbecco’s modified Eagle medium
DMSO	Dimethyl sulfoxide
DSC	Differential scanning calorimetry
EtOAc	Ethyl acetate
EtOH	Ethanol
FBS	Fetal bovine serum
HAPZ	Hydroxymethyl aripiprazole
HE	Hematoxylin and eosin
HIV	Human immunodeficiency virus
^1^H-NMR	Proton nuclear magnetic resonance
HPLC	High-performance liquid chromatography
IPA	Isopropanol
LAIs	Long-acting injectables
LCMS	Liquid chromatography-mass spectrometry
MeOH	Methanol
MRM	Multiple reaction monitoring
SCXRD	Single-crystal X-ray diffraction
TEA	Triethylamine
TGA	Thermogravimetric analysis
Tol	Toluene
PXRD	Powder X-ray diffraction
R&D	Research and Development
SEM	Scanning electron microscopy
