# Supplementary material for: Effect of Alkyl Chain Length on Physicochemical and Pharmacokinetic Performance of Aripiprazole Fatty Acid Prodrugs for Long-Acting Injectable Suspensions
Source: Pharmaceutics. 2026 Jul 24;18(8):911. doi: 10.3390/pharmaceutics18080911 (PMC13516184; doi:10.3390/pharmaceutics18080911)
Supplement: Supplementary file 1 [file pharmaceutics-18-00911-s001.zip › pharmaceutics-4421110-supplementary.pdf]

Supplementary Materials

Effect of Alkyl Chain Length on Physicochemical and Pharmacokinetic Performance of Aripiprazole Fatty Acid Prodrugs for Long-Acting Injectable Suspensions

*An Chen [1], Cong Lai [2], Hao Zhai [1], Shiyang Zhang [1], Yijing Zhang [1] and Ting Cai [1,2,]**

[a] State Key Laboratory of Natural Medicines, Department of Pharmaceutical Engineering, School of Engineering, China Pharmaceutical University, Nanjing 211198, China

[b] Department of Pharmaceutics, School of Pharmacy, China Pharmaceutical University, Nanjing 211198, China

Corresponding Authors:

*E-mail: tcai@cpu.edu.cn. Tel: 86-025-83271123.

S1. Synthesis of APZ fatty-acid prodrugs

Synthesis of hydroxymethyl aripiprazole

Anhydrous aripiprazole (9.61 g, 20.1 mmol), paraformaldehyde (2.00 g, 66.7 mmol), DBU (0.13 g, 0.8 mmol) and toluene (100 mL) were charged into a two-necked 250 mL reactor. The mixture was stirred at 40 °C for 20 h under nitrogen atmosphere. And then the reaction mixture was cooled to 4 °C and kept for 10 h. The solid was filtered from the mixture and washed with cold toluene. The product was dried at 30 °C in a vacuum oven for 6 h to obtain the hydroxymethyl aripiprazole (HAPZ), which was purified with deionized water.

Synthesis of aripiprazole fatty-acid prodrugs

Fatty acid (1.3 mmol), DCC (1.3 mmol) and dichloromethane (10 mL) were charged into a 50 mL reactor and kept under stirring for 10 min. Then, HAPZ (0.48 g, 1.0 mmol) and 4-dimethylaminopyridine (0.02 g, 0.2 mmol) were charged. The reaction mixture was kept under stirring at 30 °C for 8 h. DCC was filtered from the reaction mixture. DCM was distilled at reduced pressure until 1 mL of solvent were left in the reaction mixture. Then, 10 mL of isopropanol were added. The reaction mixture was cooled at 4 °C and kept at this temperature for 4h. The mixture was filtered and the solid was washed twice with isopropanol (15 mL). The solid was dried under vacuum at 40 °C for 12h to obtain APZ prodrug, which was purified with IPA until single melt point was observed under the polar microscope.

APZP12: ^{1}H NMR (500 MHz, Chloroform-d) δ 7.22-7.14 (m, 2H), 7.10 (d, J=8.2 Hz, 1H), 6.99 (dd, J = 7.1, 2.5 Hz, 1H), 6.71-6.56 (m, 2H), 5.95 (s, 2H), 4.01 (t, J = 6.3 Hz, 2H), 3.12 (s, 4H), 2.89 (dd, J = 8.5, 5.9 Hz, 2H), 2.72 (dd, J = 8.6, 5.9 Hz, 6H), 2.53 (d, J = 8.2 Hz, 2H), 2.39 (t, J = 7.5 Hz, 2H), 1.93-1.83 (m, 2H), 1.76 (s, 2H), 1.70-1.63 (m, 2H), 1.28 (s, 16H), 0.91 (t, J = 6.9 Hz, 3H). ES+(m/z): 660.33.

APZP16: ^{1}H NMR (500 MHz, Chloroform-d): δ7.19-7.12 (m, 2H), 7.07 (d, J = 8.0 Hz, 1H), 6.96 (dd, J = 6.4, 3.2 Hz, 1H), 6.59 (d, J = 10.8 Hz, 2H), 5.92 (s, 2H), 3.97 (t, J=6.1 Hz, 2H), 3.08 (s, 4H), 2.86 (dd, J=8.5, 5.8 Hz, 2H), 2.68 (dd, J=8.8, 5.5 Hz, 6H), 2.52 (d, J=8.1 Hz, 2H), 2.35 (t, J=7.5 Hz, 2H), 1.83 (q, J=6.6, 5.8 Hz, 2H), 1.77-1.68 (m, 2H), 1.62 (q, J=7.3 Hz, 2H), 1.24 (d, J=3.3 Hz, 24H), 0.88 (t, J=6.6 Hz, 3H). ES+(m/z): 716.40.

APZP20: ^{1}H NMR (500 MHz, Chloroform-d): δ7.20-7.10 (m, 2H), 7.06 (d, J=8.2 Hz, 1H), 6.96 (dd, J=7.2, 2.3 Hz, 1H), 6.63-6.55 (m, 2H), 5.92 (s, 2H), 3.97 (t, J=6.2 Hz, 2H), 3.09 (s, 4H), 2.86 (dd, J=8.5, 5.9 Hz, 2H), 2.68 (dd, J=8.6, 5.8 Hz, 6H), 2.51 (s, 2H), 2.35 (t, J=7.5 Hz, 2H), 1.84 (p, J=6.6 Hz, 2H), 1.73 (s, 2H), 1.63 (p, J=7.4 Hz, 2H), 1.25 (d, J=7.1 Hz, 32H), 0.88 (t, J=6.8 Hz, 3H). ES+(m/z): 772.46.

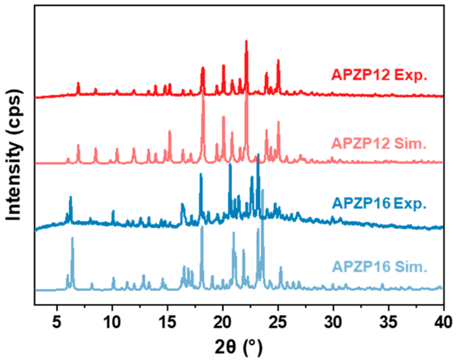

Figure S1. Experimental (Exp.) and simulated (Sim.) PXRD patterns of APZ-fatty acid prodrugs

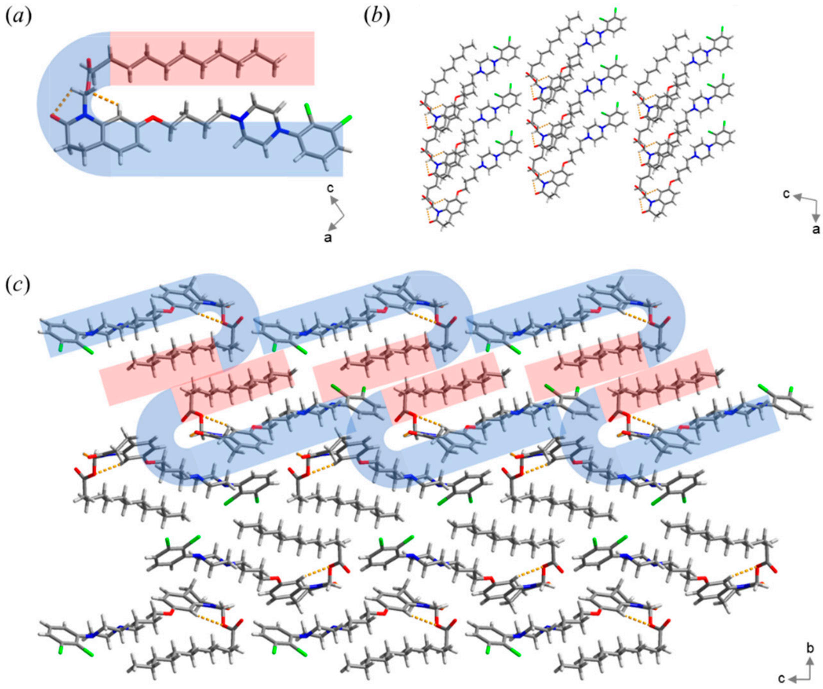

Figure S2. Crystal structure of APZP12. (a) Molecular conformation of prodrug, (b,c) molecular packing viewed along the *b*-axis (b) and the *a*-axis (c).

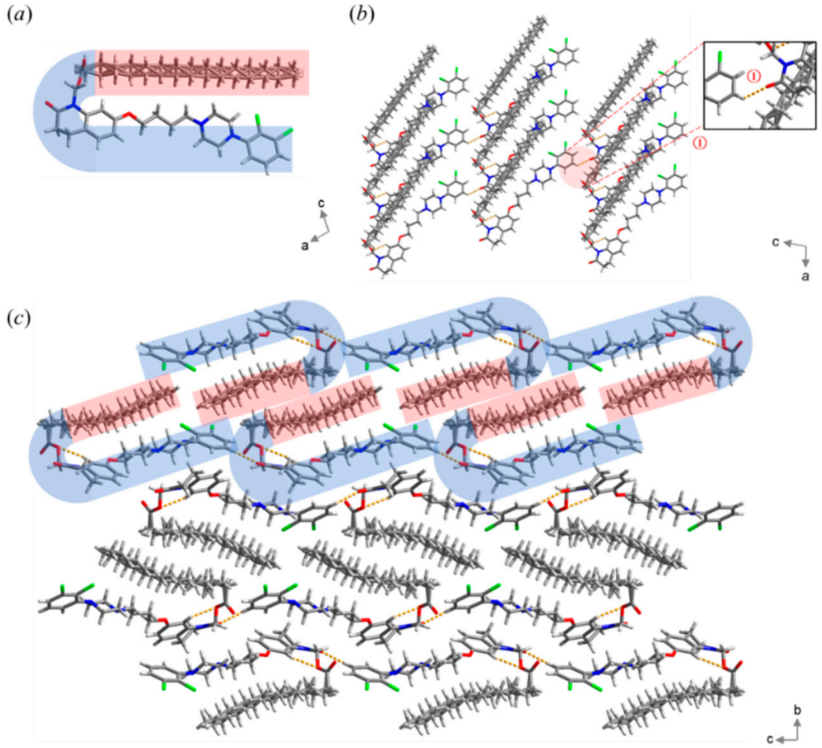

Figure S3. Crystal structure of APZP16. (a) Molecular conformation of prodrug, (b,c) molecular packing viewed along the *b*-axis (b) and the *a*-axis (c).

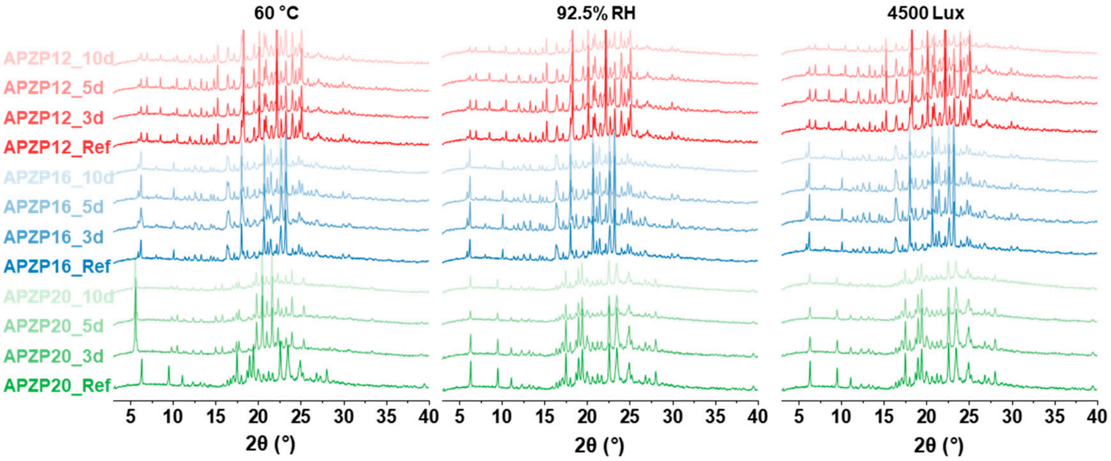

Figure S4. PXRD patterns of APZ-fatty acid prodrugs after 3, 5, and 10 days of storage under 60 °C, 92.5% RH, and 4500 lux light exposure.

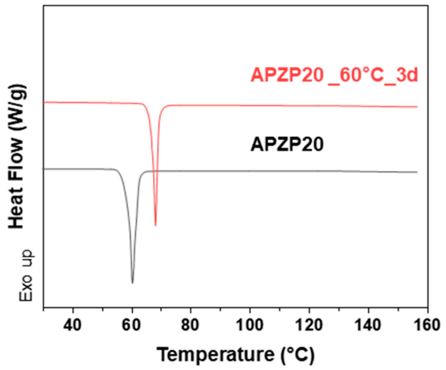

Figure S5. DSC thermogram of APZP20 after storage at 60 °C for 3 days.

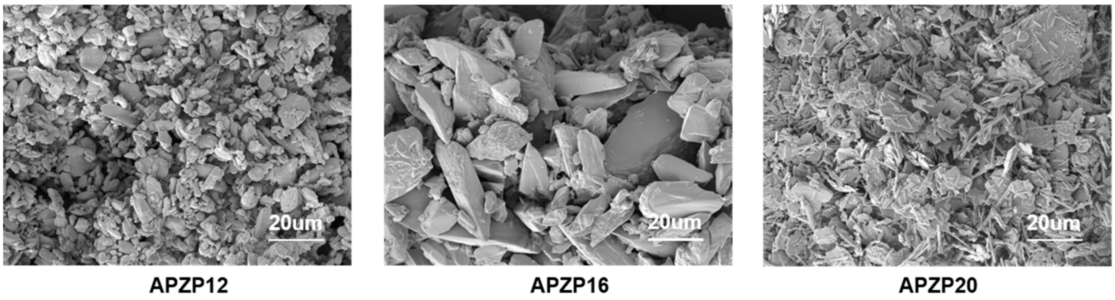

Figure S6. SEM images of APZ-fatty acid prodrugs

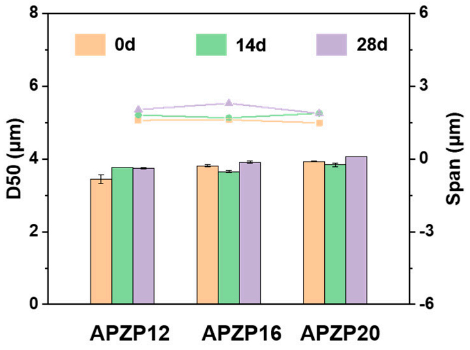

Figure S7. The D50 and Span value of APZ prodrug suspenison vs time under 4° C.

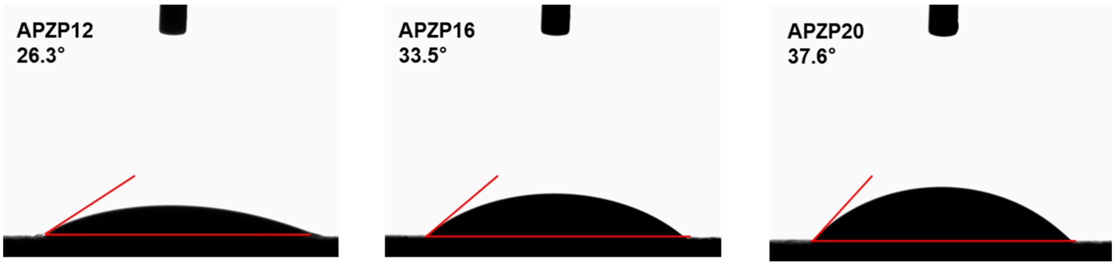

Figure S8. Contact angle of a water droplet containing 1 wt% Tween 20 on the surface of fatty acid prodrug tablets.

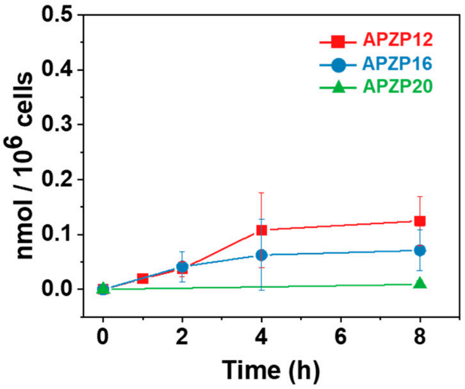

Figure S9. Intracellular APZ levels after treatment with prodrug suspensions. Missing values indicate concentration levels below the limit of detection.

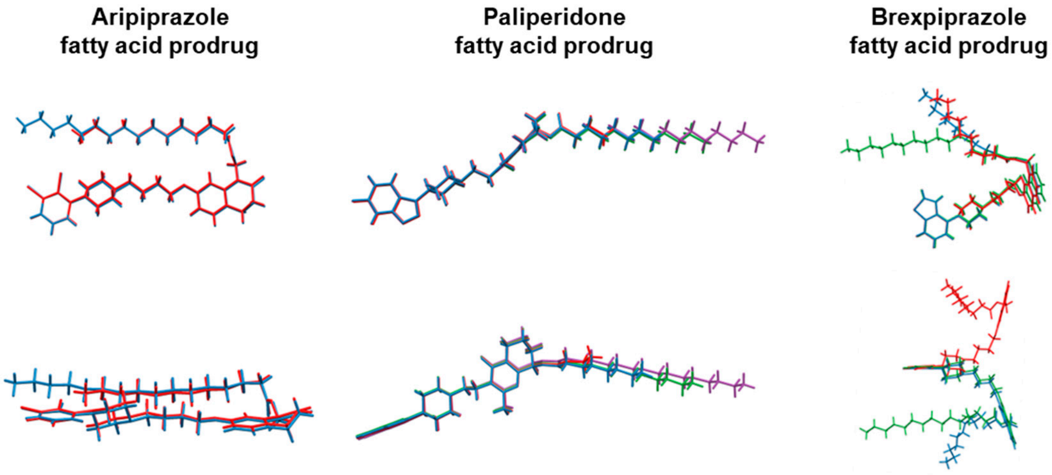

Figure S10. Comparative crystallographic data for aripiprazole, paliperidone, and brexpiprazole fatty acid prodrugs. For the aripiprazole and paliperidone series, the molecular conformations are highly consistent with negligible differences in torsion angles, which may account for the correlation between chain length and prodrug properties. In contrast, brexpiprazole prodrugs exhibit remarkable variations in both molecular conformation and torsion angles, which might explain the absence of a clear chain-length dependence in this system.

Table S1. Process parameters for the milling of APZ-fatty acid prodrug suspensions.

	APZP12	APZP16	APZP20
APZ	27.6 wt.%	29.9 wt.%	32.2 wt.%
Span 20	3.8 mg/mL	3.8 mg/mL	3.8 mg/mL
Tween 20	1.5 mg/mL	1.5 mg/mL	1.5 mg/mL
NaCl	6.1 mg/mL	6.1 mg/mL	6.1 mg/mL
Other	Sodium phosphate dibasic anhydrous, sodium phosphate monobasic were added for adjust pH		
Milling speed	800 rpm	800 rpm	800 rpm
Milling time	15 min	10 min	80 min
Bead diameter	0.8 mm	0.8 mm	0.8 mm

Table S2. HPLC parameters for the analysis of APZ-fatty acid prodrugs.

	APZP12	APZP16	APZP20
Column	Water XTERRA RP 18（3.9*150mm, 5 μm）		
Mobile phase	ACN / 0.1% formic acid and 0.1% triethylamine in water (90:10, v/v)		
Column temperature	40 °C		
Wavelength	254 nm		
Flow rate	0.5 mL/min	1.0 mL/min	1.5 mL/min

Table S3. Crystallographic parameters of APZ-fatty acid prodrugs

	APZP12	APZP16
Empirical formula	$C_{36}H_{51}Cl_2N_3O_4$	$C_{40}H_{59}Cl_2N_3O_4$
Formula weight	660.69	716.80
Crystal system	monoclinic	monoclinic
Space group	$P2_1/c$	$P2_1/c$
a (Å)	7.9327(5)	8.2378(8)
b (Å)	25.3000(17)	27.408(2)
c (Å)	18.2189(13)	17.7288(13)
α (°)	90	90
β (°)	101.123(2)	101.323(3)
γ (°)	90	90
Volume (Å^3)	3587.8	3924.93
Z	4	4
ρ_{calc} (g.cm^{-3})	1.223	1.213
R_1, wR_2 [I>2σ(I)]	0.0591, 0.1526	0.0540, 0.1182
R_1, wR_2(all data)	0.1318, 0.2056	0.0953, 0.1416
CCDC No.	2574517	2574518

Table S4. Hydrogen bond of APZ-fatty acid prodrugs

No.	D−H···A	D−H	H···A	D···A	D−H···A	Symmetry code

APZP12	1	C(23)−H(23)···O(3)	0.93	2.54	3.128(3)	121	Intra
	2	C(24)−H(24B)···O(2)	0.97	2.35	2.702(4)	100	Intra
APZP16	1	C(23)−H(23) ···O3	0.95	2.57	3.160(3)	121	Intra
	2	C(4)−H(4) ···O2	0.95	2.45	3.177(3)	133	2+x,y,1+z

Table S5. Short ring-interactions with Cg-Cg Distances < 6.0 A and Beta < 60° in APZ-fatty acid prodrugs

	No.	Cg(I)-Cg(J)	Cg-Cg	Alpha	Beta	Gamma	CgI_Perp	CgJ_Perp
APZP12	1	Cg(3)-Cg(4)	5.8581(19)	63.60(14)	23.0	78.3	1.1896(13)	5.3942(12)
	2	Cg(4)-Cg(3)	5.2242(18)	63.60(14)	28.9	70.0	1.7910(12)	4.5729(13)
APZP16	1	Cg(3)-Cg(4)	5.8979(15)	58.07(11)	27.7	79.1	1.1151(10)	5.2221(9)
	2	Cg(4)-Cg(3)	5.2418(15)	58.07(11)	32.5	67.9	1.9741(9)	4.4225(10)

Table S6. Retention time and capacity factor of APZ fatty-acid prodrugs

	t_R (min)	t_0 (min)	*k'*	*log k'*
APZP12	14.075	2.896	3.860	0.587
APZP16	44.582	2.897	14.389	1.158
APZP20	152.982	2.888	51.972	1.716

Table S7. Pharmacokinetic parameters of APZ after intramuscular administration of three APZ-fatty acid prodrug suspensions to rats (mean ± standard deviation, n=6)

	APZP12	APZP16	APZP20
$AUC_{0\text{-t}}$ (μg/L*h)	12491.4 ± 1730.5	12268.4 ± 2352.6	5658.2 ± 1818.1
$AUC_{0\text{-}\infty}$ (μg/L*h)	12879.8 ± 1982.1	12660.2 ± 2350.5	6613.7 ± 2460.4
$t_{1/2}$ (h)	126.4 ± 61.3	140.1 ± 44.1	115.2 ± 36.3
CL (L/h/kg)	3.30 ± 0.5	3.40 ± 0.6	7.45 ± 3.8
C_{max} (μg/L)	52.4 ± 14.3	44.4 ± 6.3	25.8 ± 12.6

Table S8. Research on the relationship between fatty acid alkyl chain length and prodrug properties

Parent drugs	Fatty-acid prodrugs	Fatty-acid prodrugs with lowest aqueous solubility	Fatty-acid prodrugs with highest melting point	Fatty-acid prodrugs selected for subsequent researsrch	Fatty-acid prodrugs with crystal structure	Reference

Apomorphine (AMP)	DLA (C12), DPA (C16)	Not provided	Not provided	Not provided	Not provided	[1]
Aripiprazole (APZ)	APZ-P12, APZ-P16, APZ-P20			$APZP_{12}$	Yes	This research
Brexpiprazole (BPZ)	BPZO (C8), BPZD (C10), BPZL (C12), BPZP (C16)	BPZL (C12)	BPZL (C12)	BPZL (C12)	Yes	[2]
Cabotegravir (CAB)	MCAB (C14), M2CAB (C18), M3CAB (C22)	M3CAB (C22)	Not provided	M2CAB (C18)	Not provided	[3]
Didanosine (DDI)	CA-DDI (C10), MA-DDI (C14), SA-DDI (C18)	Not provided	Not provided	SA-DDI (C18)	Not provided	[4]
Dolutegravir (DTG)	MDTG (C14), M2DTG (C18), M3DTG (C22)	M2DTG (C18)	MDTG (C14)	M2DTG (C18)	Not provided	[5]
Entecavir (EV)	EV-D (C14), EV-P (C16), EV-S (C18)	EV-P (C16)	Not provided	EV-P (C16)	Not provided	[6]
Naltrexone (NTX)	ACE-NTX (C2), PROP-NTX (C3), BUT-NTX (C4), VAL-NTX (C5), HEX-NTX (C6), HEP-NTX (C7)	HEP-NTX (C7)	PROP-NTX (C3)	Not provided	Not provided	[7]
Nalmefene (NMF)	C8, C10, C12, C16	Not provided	Not provided	C12	Not provided	[8]
Olanzapine (OLZ)	3ba-3bj (C2-C20)	3bf (C12)	3bj (C20)	Not provided	Yes	[9]
Paliperidone (PAL)	PC4, PC8, PC12, PC16	PC16 (C16)	PC16 (C16)	PC16 (C16)	Yes	[10]
Rilpivirine (RPV)	M1RPV (C7), M2RPV (C12), M3RPV (C14), M4RPV (C18)	Not provided	Not provided	M3RPV (C14)	Not provided	[11]
Zidovudine (AZT)	C2-AZT, C3-AZT, C4-AZT, C6-AZT, C8-AZT, C10-AZT, C12-AZT, C14-AZT, C16-AZT, C18-AZT	Not provided	C16-AZT	C2-AZT, C10-AZT, C18-AZT	Not provided	[12]
7-ethyl-10	SN-38-C2, SN-	Not	Not	Not	Not	[13]

hydroxycamptothecin (SN-38)	38-C6, SN-38-C10, SN-38-C14, SN-38-C18,	provided	provided	provided	provided	

1. Borkar, N.; Li, B.; Holm, R.; Håkansson, A. E.; Müllertz, A.; Yang, M.; Mu, H., Lipophilic prodrugs of apomorphine I: Preparation, characterisation, and in vitro enzymatic hydrolysis in biorelevant media. *Eur J Pharm Biopharm* **2015,** 89, 216-223.
2. Wang, J.; Liu, J.; Ding, J.; Li, Q.; Zhao, Y.; Gao, D.; Su, K.; Yang, Y.; Wang, Z.; He, J., Creation of a ready-to-use brexpiprazole suspension and the inflammation-mediated pharmacokinetics by intramuscular administration. *Eur J Pharm Biopharm* **2023,** 189, 189-201.
3. Kulkarni, T. A.; Bade, A. N.; Sillman, B.; Shetty, B. L. D.; Wojtkiewicz, M. S.; Gautam, N.; Hilaire, J. R.; Sravanam, S.; Szlachetka, A.; Lamberty, B. G.; Morsey, B. M.; Fox, H. S.; Alnouti, Y.; McMillan, J. M.; Mosley, R. L.; Meza, J.; Domanico, P. L.; Yue, T.-Y.; Moore, G.; Edagwa, B. J.; Gendelman, H. E., A year-long extended release nanoformulated cabotegravir prodrug. *Nat Mater* **2020,** 19, (8), 910-920.
4. Chen, H.; Wang, G.; Sun, L.; Zhang, H.; Sun, M.; Sun, J.; Shang, L.; Luo, C., Regulating the alky chain length of fatty acid-didanosine prodrugs and evaluating its role in albumin binding. *Drug Drug Deliv Transl Res* **2018,** 8, (1), 21-31.
5. Deodhar, S.; Sillman, B.; Bade, A. N.; Avedissian, S. N.; Podany, A. T.; McMillan, J. M.; Gautam, N.; Hanson, B.; Dyavar Shetty, B. L.; Szlachetka, A.; Johnston, M.; Thurman, M.; Munt, D. J.; Dash, A. K.; Markovic, M.; Dahan, A.; Alnouti, Y.; Yazdi, A.; Kevadiya, B. D.; Byrareddy, S. N.; Cohen, S. M.; Edagwa, B.; Gendelman, H. E., Transformation of dolutegravir into an ultra-long-acting parenteral prodrug formulation. *Nat Commun* **2022,** 13, (1), 3226.
6. Ho, M. J.; Lee, D. R.; Im, S. H.; Yoon, J. A.; Shin, C. Y.; Kim, H. J.; Jang, S. W.; Choi, Y. W.; Han, Y. T.; Kang, M. J., Microsuspension of fatty acid esters of entecavir for parenteral sustained delivery. *Int J Pharm* **2018,** 543, (1), 52-59.
7. Stinchcomb, A. L.; Swaan, P. W.; Ekabo, O.; Harris, K. K.; Browe, J.; Hammell, D. C.; Cooperman, T. A.; Pearsall, M., Straight-Chain Naltrexone Ester Prodrugs: Diffusion and Concurrent Esterase Biotransformation in Human Skin. *J Pharm Sci* **2002,** 91, (12), 2571-2578.
8. Gaekens, T.; Guillaume, M.; Borghys, H.; De Zwart, L. L.; de Vries, R.; Embrechts, R. C. A.; Vermeulen, A.; Megens, A. A. H. P.; Leysen, J. E.; Herdewijn, P.; Annaert, P. P.; Atack, J. R., Lipophilic nalmefene prodrugs to achieve a one-month sustained release. *J Control Release* **2016,** 232, 196-202.
9. Blumberg, L. C.; Zeidan, T. A.; Maddaford, A.; Warren, N. C.; Hutchison, P., Novel N-5-(acyloxyalkoxy)carbonyl prodrugs of olanzapine with physicochemical properties for extended-release. *RSC Adv* **2013,** 3, (37), 16270-16278.
10. Chen, A.; Cai, P.; Peng, Y.; Guo, M.; Su, Y.; Cai, T., The role of alkyl chain length in the melt and solution crystallization of paliperidone aliphatic prodrugs. *IUCrJ* **2024,** 11, (1), 23-33.
11. Hilaire, J. R.; Bade, A. N.; Sillman, B.; Gautam, N.; Herskovitz, J.; Dyavar Shetty, B. L.; Wojtkiewicz, M. S.; Szlachetka, A.; Lamberty, B. G.; Sravanam, S.; Fox, H. S.; Alnouti, Y.; Dash, P. K.; McMillan, J. M.; Edagwa, B. J.; Gendelman, H. E., Creation of a long-acting rilpivirine prodrug nanoformulation. *J Control Release* **2019,** 311-312, 201-211.

12. Kawaguchi, T.; Ishikawa, K.; Seki, T.; Juni, K., Ester prodrugs of zidovudine. *Journal of Pharmaceutical Sciences* **1990,** 79, (6), 531-533.
13. Koseki, Y.; Ikuta, Y.; Kamishima, T.; Onodera, T.; Oikawa, H.; Kasai, H., Drug Release is Determined by the Chain Length of Fatty Acid-Conjugated Anticancer Agent as One Component of Nano-Prodrug. *Bull Chem Soc Jpn* **2016,** 89, (5), 540-545.
